# Farm resilience to climatic risk. A review

**DOI:** 10.1007/s13593-024-00998-w

**Published:** 2025-02-06

**Authors:** Valentin Pret, Gatien N. Falconnier, François Affholder, Marc Corbeels, Regis Chikowo, Katrien Descheemaeker

**Affiliations:** 1 AIDA, Univ Montpellier, CIRAD, Montpellier, France; 2 CIRAD, UPR AIDA, Harare, Zimbabwe; 3 Plant Production Systems Group, Wageningen University & Research, Wageningen, Netherlands; 4 International Maize and Wheat Improvement Centre (CIMMYT)-Zimbabwe, Harare, Zimbabwe; 5 Faculdade de Agronomia e Engenharia Florestal, Universidade Eduardo Mondlane, Maputo, Mozambique; 6 CIRAD, UPR AIDA, Nairobi, Kenya; 7 International Institute of Tropical Agriculture, Nairobi, Kenya; 8 Department of Plant Production Sciences and Technologies, University of Zimbabwe, Harare, Zimbabwe

**Keywords:** Food security, Climate change, Smallholder, Farm system, Resilience assessment

## Abstract

Increasing our understanding of farm resilience drivers to climate-related risks is critical for designing innovative farm systems, especially for smallholders that are highly vulnerable to climatic hazards and expected to follow a pathway toward sustainable development. However, the literature is fragmented on the concepts and methods to measure farm resilience. Moreover, quantitative assessments of options to enhance farm resilience to climate risks are scarce. Resilience can be defined as the ability of a system to recover, reorganize and evolve following external stresses and disturbances. Such definition can be applied to farm systems. In this study, we systematically reviewed how changes in resilience-enhancing attributes (reserves, openness, modularity, tightness of feedbacks and diversity) impacted farm performance and resilience to climate-related risks, with a specific attention to smallholder farms. Our analysis showed that reviewed studies assessed farm resilience using the agricultural and economic dimensions of performance, often excluding the socio-environmental dimensions. To assess performance, the average value of indicators was most commonly employed, sometimes combined with variability metrics or the probability of exceeding a critical threshold. Improving one resilience attribute increased one dimension of farm performance for a given metric in most of the studies, but some studies showed the opposite effect. The lack of comprehensive assessments exploring different attributes and their impact on several dimensions of performance using diverse metrics prevents a robust conclusion on how to improve farm resilience to climate-related risks. Therefore, we recommend to pay more attention to quantitative assessments of farm resilience, including a systematic investigation of the temporal variability of performance and the socio-environmental dimensions of performance. Finally, we emphasize the need to focus on the recovery of smallholder farms after a disturbance, with the goal of achieving growth in farm performance rather than simply reverting to their current state of food insecurity and poverty.

**Supplementary Information:**

The online version contains supplementary material available at 10.1007/s13593-024-00998-w.

**Contents**

- 1. Introduction
- 2. Methodology
- 2.1 Literature query and article selection
- 2.2 Farm attributes of resilience
- 2.3 Study categorization
- 3. Definition and assessment of farm resilience
- 3.1 Definition of resilience
- 3.2 Scope and methods to assess farm resilience
- 3.3 Dimensions and metrics to quantify farm performance
- 3.4 Attributes of farm resilience
- 4. Impact of resilience attributes on farm performance
- 4.1 Relation between a single resilience attribute and farm performance
- 4.2 Relation between combined resilience attributes and farm performance
- 4.3 Studies that assessed multiple dimensions and metrics of farm performance
- 4.4 A lack of comprehensive assessments of the impact of resilience attributes on farm performance
- 5. Towards a comprehensive assessment of farm resilience tailored to smallholder farms
- 5.1 Recommendations for quantitative assessment of farm resilience based on farm performance
- 5.2 Mechanistic models to assess farm resilience
- 5.3 Simply bouncing back is not an option for smallholder farms
- 6. Conclusion
- Acknowledgement
- References

## Introduction

**Fig. 1:**
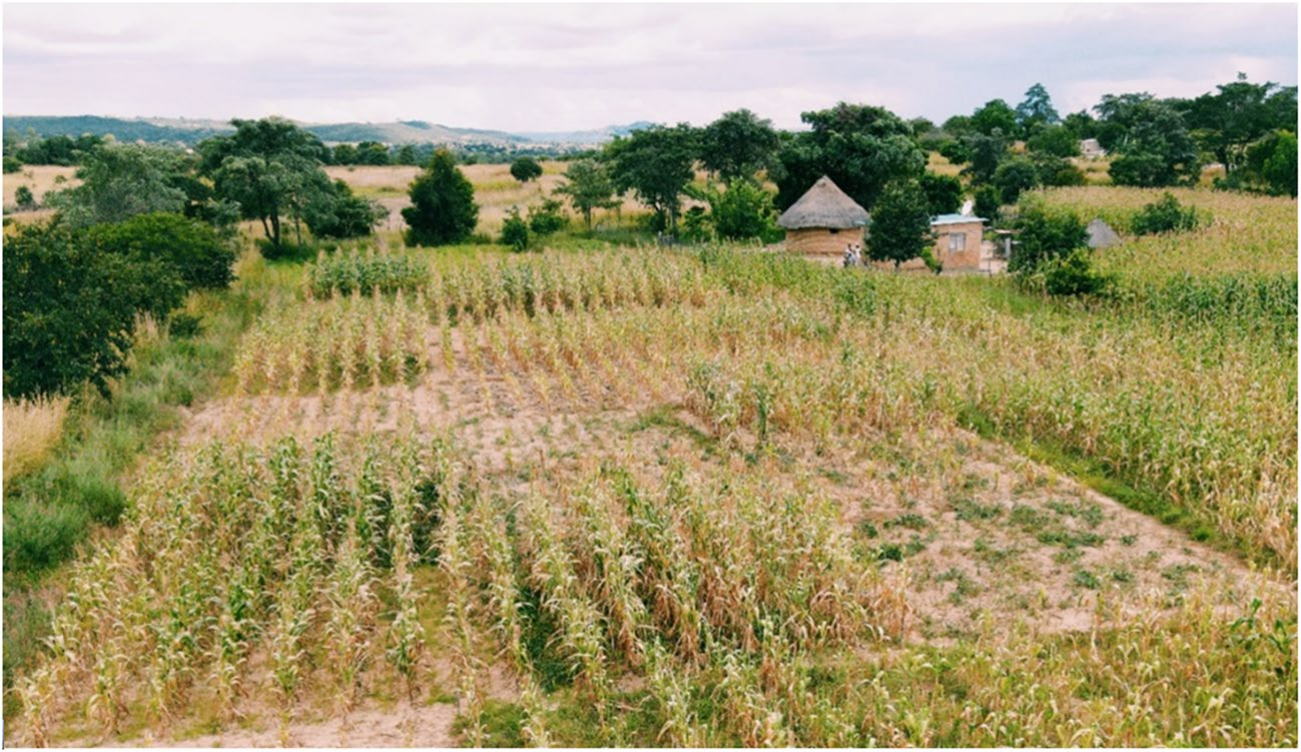
Illustration of the impact of a dry spell on rainfed maize plants during grain filling stage in a smallholder farm in southern Africa. Maize water stress, recognizable by advanced senescence of leaves (*i.e.*, dried and yellowish), affects grain filling and thus grain yield with direct consequences on farm food security (photograph: ©Cirad)

Climate change is associated with an increase in global temperature and changes in seasonality, distribution and amount of precipitation, which increases uncertainties related to food production. Future climate projections also indicate an increase in extreme weather events, such as droughts, with negative impacts on crop yield, livestock production and food security (Olesen and Bindi 2002; Thornton et al. 2014; IPCC 2023). There are nearly 570 million farms worldwide, of which the vast majority are smallholder farms characterized by land sizes of less than two hectares (Lowder et al. 2016). These smallholder farms provide a substantial share of the globally produced food calories, with estimates of the contribution ranging between 30% (Ricciardi et al. 2018) and 55% (Samberg et al. 2016). Smallholder farmers are highly vulnerable to climate-related risks, largely due to their heavy reliance on rainfed agriculture (Figure 1), their limited access to production inputs, and the lack of infrastructure and governmental support (Challinor et al. 2007; Descheemaeker et al. 2016). Intensifying crop production of smallholder systems is a way to improve both food security and farm income, for example through the use of mineral fertilizer in sub-Saharan Africa (Vanlauwe et al. 2023; Falconnier et al. 2023a), but it comes at the cost of increased risks related to inter-annual climate variations compared to current low-intensity cropping practices (Keating et al. 1991; Affholder 1995; Rötter et al. 1997; de Rouw 2004; Falconnier et al. 2020). Improving food security and adaptation to climate change of smallholder farms are therefore high on the research and development agenda.

Originally applied to ecological systems to analyze their complex dynamics in the face of change and shocks (Holling 1973), resilience was extended to socio-ecological systems (Walker et al. 2004; Folke et al. 2010) and agroecosystems (Darnhofer et al. 2010). Resilience has been commonly defined as the ability of a system to bounce back to a stable state after a disturbance (Davoudi et al. 2012; Scott 2013), yet this definition has been criticized for its narrow scope (Scott 2013; Dixon and Stringer 2015; Walker 2020). Resilience of smallholder farms and the adaptive capacity of their farmers are often presented as particularly high compared to other types of farm systems and farmers, but also commonly judged as insufficient to cope with climate change, especially in a semi-arid climate (Jellason et al. 2021). However, the application of the concept of resilience to the context of smallholder farms in low- and middle-income countries requires a careful reflection on the kind of resilience that is desired. Barrett and Constas (2014) and Chaigneau et al. (2022) highlighted the paradox of smallholder farms being resilient to shocks, but being trapped in poverty and failing to achieve well-being. In such situations, bouncing back to a stable state is not desirable and would actually conflict with several sustainable development goals of the United Nations, starting with SDG1 and 2 (respectively “No Poverty” and “Zero hunger”). Considering resilience as the “ability for a farm to maintain a trajectory of development to avoid chronic poverty” (Barrett and Constas 2014) seems more suitable to the smallholder context. In the present article we choose to follow Diserens et al. (2018) defining resilience as “the ability of a system to recover, reorganize and evolve following external stresses and disturbances”, as more general than the statement from Barrett and Constas (2014) cited above, but still relevant for the context of smallholder systems in low- and middle-income countries.

Cabell and Oelofse (2012) proposed 13 indicators, related to ecological, social and economic dynamics of the farm (*e.g.,* functional diversity, degree of coupling with local natural capital), to assess farm resilience. The authors emphasized the fact that measuring resilience is "aiming at a moving target" due to the complexity of agroecosystems which are characterized by nonlinear feedback mechanisms that occur at multiple spatial and temporal scales, leading to unpredictable dynamics and patterns of abrupt changes. Hence, resilience assessment of agricultural systems is challenging (Urruty et al. 2016; Douxchamps et al. 2017; Pimm et al. 2019). Meuwissen et al. (2019) developed an integrated framework to assess resilience of farming systems revolving around five resilience-enhancing attributes (namely reserves, openness, modularity, tightness of feedbacks and diversity), in line with the indicators defined by Cabell and Oelofse (2012). This framework has proven useful in guiding the design of more resilient agricultural systems in the Netherlands (Reidsma et al. 2023). More precisely, the particular focus of these authors was on the interactions between the farms and the ecological, social, economic and political systems of a region. Yet, the question of resilience to climate applied to smallholder farms along a trajectory of development may need a stronger focus on the contribution of within-farm interactions to resilience (*e.g.,* crop and livestock interactions), and hence on the farm system, rather than the larger farming system (Giller 2013).

The causal links between resilience attributes and actual resilience are often assumed and often based on qualitative expert assessment and perception rather than quantified data, especially in the smallholder context (Paas et al. 2021; Alary et al. 2022). Experts like farmers and other local actors (food processors, agro-dealers, decision-makers) have a deep understanding of their working environment, yet their analysis of the issues and processes at stake can be incomplete (Van Asten et al. 2009), often leading to biased perceptions of what drives resilience. In order to accurately and credibly assess current and future performance of farm systems and their resilience, qualitative methods, including methods relying on expert analysis and perception of resilience, need to be complemented with quantitative ones (Clark et al. 2011). However, there are few comprehensive studies that quantify the links between resilience attributes and farm performance and overall resilience to climate-related risks (*e.g.,* Barber et al. 2020). Studies assessing smallholders’ resilience on a trajectory of development with quantitative methods are even scarcer (*e.g.,* Nienkerke et al. 2024). In a recent review, Dardonville et al. (2021) analyzed the quantitative links between farm performance dynamics (including resilience) and explanatory factors of these dynamics (including resilience attributes) but focused on the case of non-smallholder farms in the temperate climate of a high-income country. To the best of our knowledge, there has been no systematic review, including smallholder systems in low- and middle-income countries, aiming at understanding generic quantitative links between resilience attributes and their impact on farm performance and resilience to climate-related risk. Such comprehensive assessment is critical in guiding the design of resilient farm systems to effectively address the challenges of weather variability and climate change.

In this study, we focused on climate-related risks as a major disturbance to farming, with an emphasis on the farm system level. We aimed at answering the following research question through a systematic review of the literature: What are the key resilience attributes that contribute to increase farm resilience to climate-related risk? By doing so, we answered the following sub-questions: i) What are the current methods to assess resilience at farm level? And ii) How do these methods inform the quantitative assessment of resilience? In the discussion, we explored the implications of our findings for the assessment of resilience to climate-related risk. We paid particular attention to the context of smallholder farms in in low- and middle-income countries in order to account for their high vulnerability to climatic hazards, their current limited access to resources and unfavorable institutional and economic environment and the need for them to follow a trajectory towards sustainable development.

## Methodology

### Literature query and article selection

We performed a systematic review of the literature following the PRISMA guidelines (Moher et al. 2010). We used the Web of Science (WOS) Core Collection and Scopus databases, starting from the beginning of the collections to August 2024. We targeted peer-reviewed journal articles written in English, with online available version of the publication. Our research query was structured in three sections as followed:

$$\begin{aligned}&\text{TOPIC }\left(\text{WOS}\right) /\text{ TITLE}-\text{ABS}-\text{KEY }\left(\text{Scopus}\right)\\ &= \left(\text{risk OR resilience OR vulnerability}\right)\text{ AND }(''\text{climate}\;\\ &\mathrm{change}''\text{ OR }''\text{climate variability}'')\text{ AND }(''{\text{farm}}^{*}\text{ system}'').\end{aligned}$$

Climate risk and farm resilience were our two main entry points for this study. Vulnerability was added as a key feature of smallholder farms and as a foundation of risk conceptualization (Brooks 2003). By including "risk" and "vulnerability" in our query, we aimed to use the methods and results from a broader body of literature to inform the impact of resilience attributes on farm resilience to climate risk. As such, we also ensured not to overlook studies that were related to resilience even if the authors were not using that specific term. Numerous other concepts are related to farm resilience such as stability, robustness, adaptability or transformability, which can be linked to the dynamics and capacities a system displays when facing a disturbance (Urruty et al. 2016; Meuwissen et al. 2019; Dardonville et al. 2021; van der Lee 2022). We did not include these terms in our research query because we focused on assessing the drivers of farm resilience to climate-related risk rather than characterizing the dynamic responses of farms to a disturbance. "Climate change" and "climate variability" were included to restrain the search to climate-related hazards. We also restrained the scope of the literature review to farm systems. Building up on the work of Dardonville et al. (2021), we extended our review beyond the temperate climate zone and included studies from any region of the world. In addition, we focused on the farm level but including any size of farms. This broad focus aimed to capture a comprehensive range of approaches and methods to inform how the resilience of smallholder farms to climate-related risks can be meaningfully assessed. Among the explanatory factors of resilience, we focused on the five resilience-enhancing attributes described by Meuwissen et al. (2019). We encompassed all types of methods to assess resilience, including studies that assess resilience through qualitative farmer perceptions and resilience attributes, rather than solely relying on quantitative evaluations of farm performance.

**Fig. 2:**
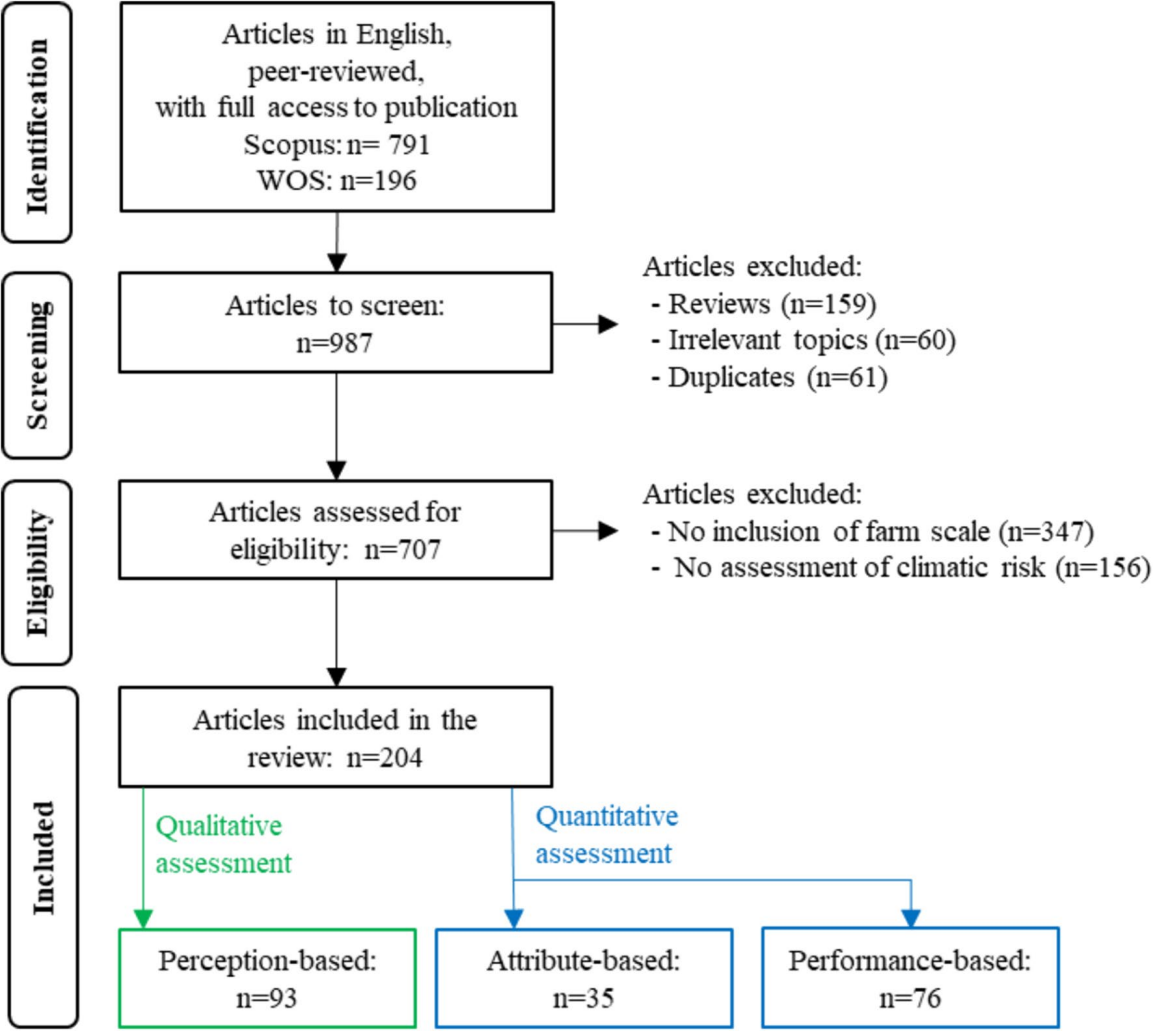
Description of the steps followed to select the articles included in the systematic review according to the PRISMA guidelines for a systematic review (Moher et al. 2010) and to organize them in three bodies of the literature: perception-based body, attribute-based body and performance-based body (qualitative assessments of resilience are in green and quantitative assessments are in blue)

From the research query, we identified 987 papers from which we removed reviews (*n*=159), papers dealing with irrelevant topics (*n*=60, indicated by the Scopus subject area or the Web of Science categories, *e.g.,* Arts and Humanities) and duplicates (*n*=61) (Figure 2). Based on the titles and abstracts of the articles, we applied two criteria of eligibility. First, studies dealing solely with crop-, field-, landscape- or regional level topics (*n*=347, *e.g.,* field measurements in controlled experimental conditions) were excluded. Second, studies not assessing a climate hazard (*n*=156, *e.g.,* dealing solely with economic or political hazards, conceptual frameworks) were excluded. Figure 2 only reports one of these eligibility criteria when both criteria were not met to include a paper. The final set of papers included for our analysis consisted of 204 papers.

### Farm attributes of resilience

**Fig. 3:**
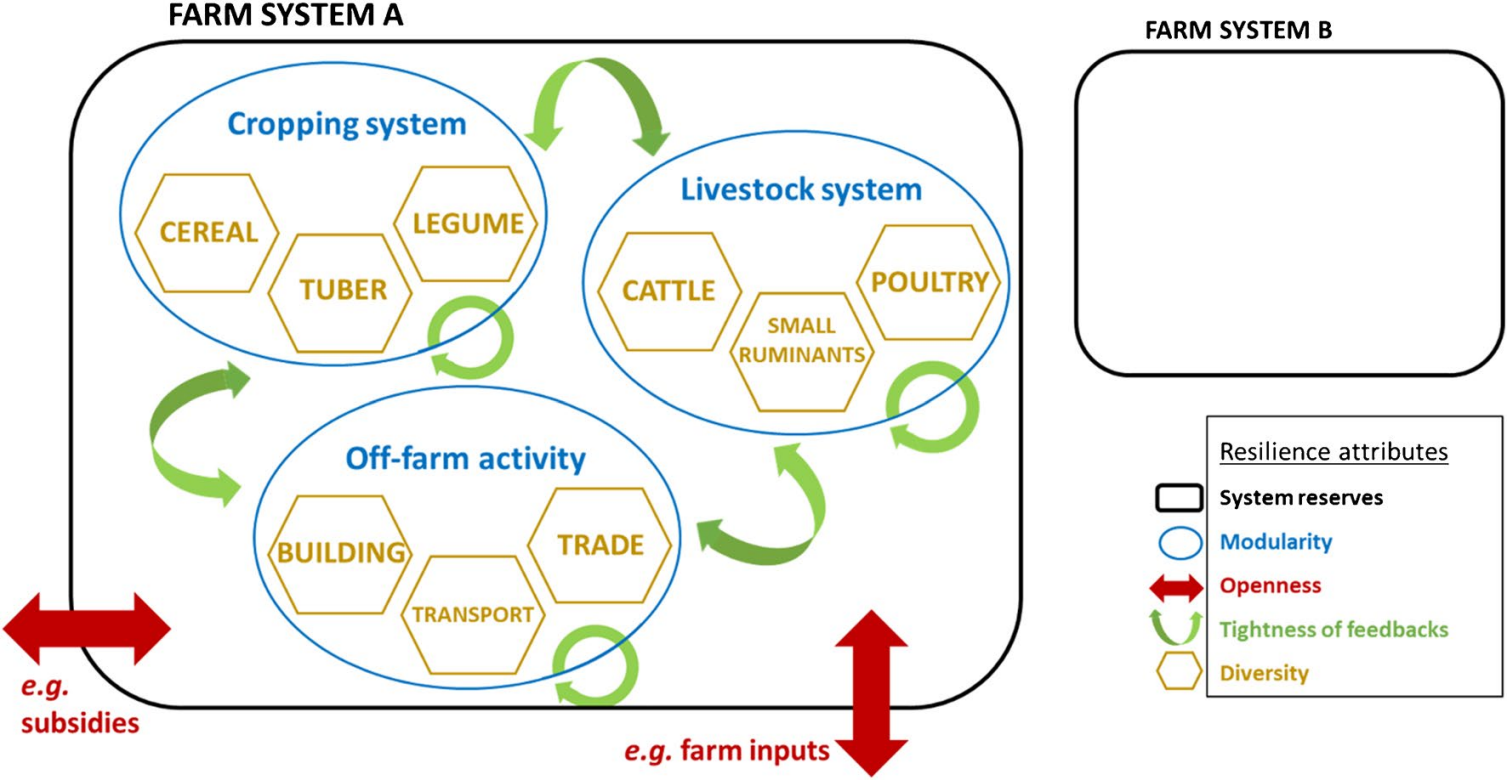
Resilience attributes and their interconnections at farm level. Adapted from Meuwissen et al. (2019). Black squares represent system reserves for farm system **A** and **B** (farm reserves are larger for system A than B). Blue circles represent the different modules and/or activities for a given farm system, with green arrows representing the interconnections between and within them. Red arrows represent the interconnection between the farm system and the external environment. Yellow hexagons represent the diversity of sub-components for a given module

Meuwissen et al. (2019) identified five attributes to assess resilience within a "local network of farms and other actors formally and informally interacting in a specific agroecological context". They use the term "farming system" to describe this, corresponding to that of the “agrarian system” (Cochet 2012). Here, we focus on the farm system, defined as a decision making system that encompasses various modules of production (*e.g.,* cropping and livestock systems) interacting with its biophysical and socio-economic environment (Fresco and Westphal 1988). The study by Meuwissen et al. (2019), by being centered on a higher hierarchy level than the farm system, did not explicitly consider within-farm interactions. Hence, aiming specifically at reviewing farm-level resilience, we adapted the five resilience attributes to be centered at the farm level, thereby integrating the interactions within the farm. The resilience attributes include system reserves (Biggs et al. 2012; Kerner and Thomas 2014), openness (Carpenter et al. 2012), modularity (Carpenter et al. 2012), tightness of feedbacks (Walker and Salt 2006) and diversity (Kerner and Thomas 2014) (Figure 3). System reserves encompass the farm resource stocks (*e.g.,* natural or economic assets) that help respond to stress and shocks and are measured by the quantity of a given stock. Openness refers to the connectivity and flows between the external environment and the farm entity (*e.g.,* governmental subsidies, access to inputs). It is measured either as a binary variable (*e.g.,* access or no access to weather insurance) or by the magnitude of the flow (*e.g.,* amount of mineral fertilizer). Modularity is the internal subdivision of the farm into interconnected modules of activities (*e.g.,* cropping system, livestock system and off-farm activities) and is measured by the number of modules within a farm. Tightness of feedbacks is the internal connectivity and flows between the farm modules (*e.g.,* nutrient transfers, labor flows) and within a farm module (*e.g.,* crop residues used as mulch on cropland). It is measured by the number of links between modules or the number of feedback loops within a module. Diversity is the agrobiodiversity within a module (*e.g.,* crop diversity, livestock diversity) and is measured by the degree of species richness within a module (*e.g.,* a farm cultivating three crops is more diverse than a farm cultivating two crops). We did not include species functional traits to describe diversity because their benefits were not detailed in the reviewed studies. Their inclusion would have required assumptions on the ecological interactions related to these traits that were out of the scope of our review.

### Study categorization

**Fig. 4:**
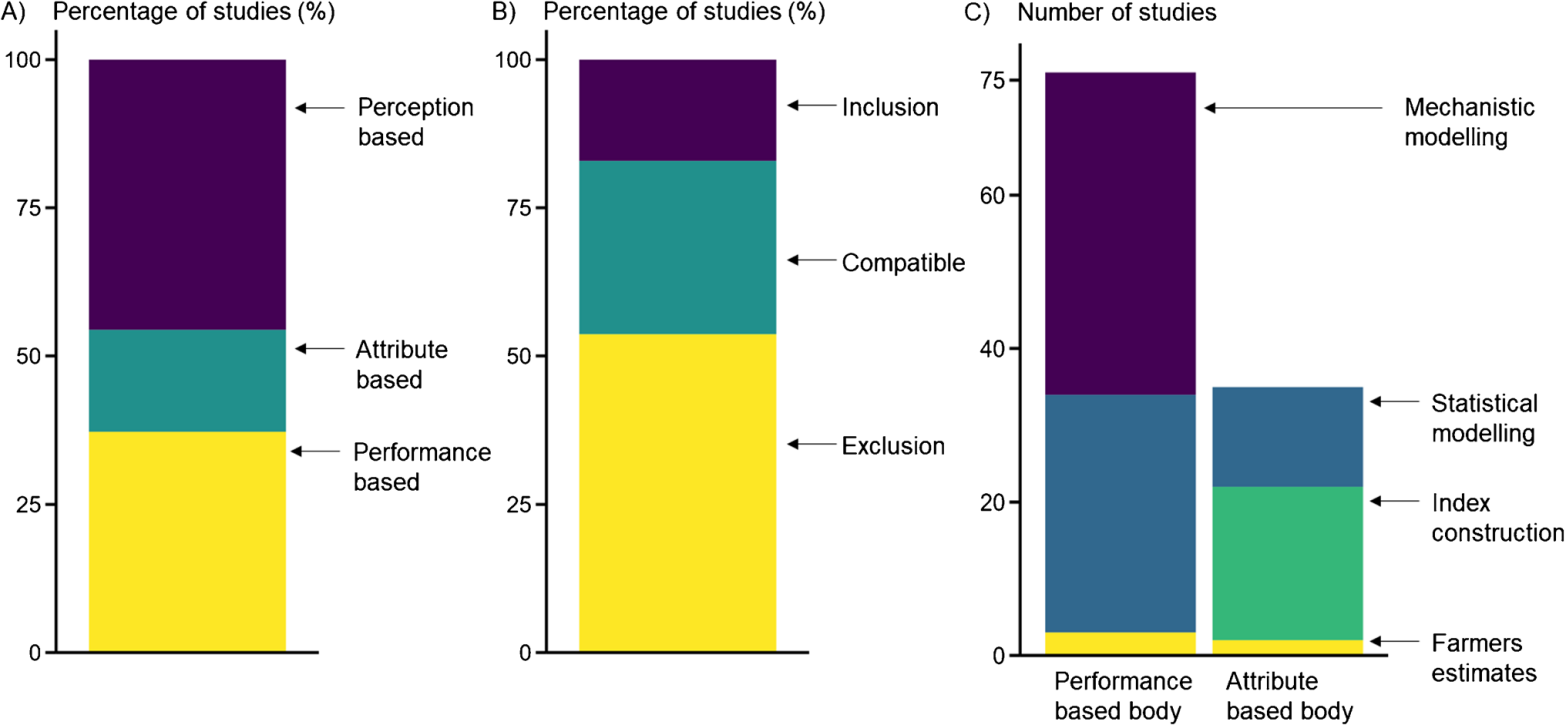
Distribution of A) the 204 studies from the systematic review in the three bodies of literature. Perception-based body: qualitative assessment of farm resilience (*n*=93); Attribute-based body: quantitative assessment of farm resilience attributes (*n*=35); Performance-based body: quantitative assessment of farm resilience attributes and their impact on farm performance (*n*=76); B) the 41 definitions of farm resilience according to their degree of integration of a trajectory of farm development: inclusion (*n*=7), compatible (*n*=12), exclusion (*n*=22); C) the quantification methods used by the attribute-based and the performance-based bodies of the literature

The 204 papers of the systematic review were grouped into three bodies of the literature (Figure 4A). The studies utilizing qualitative assessments based on farmers' perceptions of resilience formed the *"perception-based body"* (93 studies). Studies that only quantified resilience attributes formed the *"attribute-based body"* (35 studies) and those that quantified both resilience attributes and farm performance indicators formed the *"performance-based body"* (76 studies).

We discriminated studies operating in "high-income countries" (*n*=52) or in "low- and middle-income countries" (*n*=151) using the United Nations classification (UNCTAD 2022). In the case of the low- and middle-income countries, we categorized the farms as smallholder systems (*n*=142), after ensuring that these studies did not include farms with large industrial plantations like cocoa, coffee or sugarcane. The only exceptions were eight studies conducted in Latin America, Southern Africa and Central Asia, which featured large-scale farms (Simelton et al. 2013; Bobojonov and Aw-Hassan 2014; Djanibekov and Khamzina 2016; Paul et al. 2017; Tibesigwa et al. 2017; Bilotto et al. 2019, 2021; Carauta et al. 2021). For these eight studies and all studies conducted in high-income countries, the farms were defined as non-smallholder farms (*n*=59). Three studies included both non-smallholder and smallholder farms in their analysis (Toni and Holanda 2008; Le Goff et al. 2022; Abdollahzadeh et al. 2023), among which one study compared mixed crop-livestock systems between non-smallholder farms in Switzerland and smallholder farms in Uganda (Le Goff et al. 2022). When the authors of a study stated in their objectives to assess resilience, we recorded the definition of resilience used by the authors and we categorized these studies as part of the resilience assessment literature, as opposed to the studies belonging to the climatic risk and vulnerability assessment literature.

For each study of the *attribute-based* and the *performance-based* bodies of the literature, we identified i) the resilience attributes and ii) the indicators of farm performance used by the authors. We also classified the methods used to quantify the attributes of resilience and the impact of a change in resilience attributes on farm performance into four categories, namely mechanistic modelling, statistical modelling, index construction or farmer estimates (Table 1). We further distinguished between studies assessing farm performance under current climate and those examining farm performance with future climate projections. Finally, we reported when stakeholders were engaged in either the identification or quantification of attributes and indicators.

**Table 1:** Description and definition of categories and sub-categories used by the authors of this review to categorize studies systematically reviewed according to which type of body of literature they belonged to. Resilience attributes were adapted from Meuwissen et al. (2019)

Bodies of literature	Categories of information	Sub-categories of information	Definition	Number of studies
Perception-based, attribute-based and performance-based	Development category	Low- and middle-income countries		151
		High-income countries		52
		Low-, middle- and high-income countries		1
	Farm type	Smallholder		142
		Non-smallholder		59
		Smallholder and non-smallholder		3
Attribute-based and performance-based	Resilience attributes	System reserves	Resource stocks (*e.g.* natural, economic) to which a system has access when responding to stress and/or shocks	32
		Openness	Connectivity and flows between external systems to the farm and the farm itself (*e.g.* governmental subsidies, access to inputs)	43
		Modularity	Internal division of the system in independent but connected modules of activities (*e.g.* cropping system, livestock system, off-farm activities)	27
		Tightness of feedbacks	Internal connectivity and flows between farm modules (*e.g.* nutrient transfer, labour flows) and within a farm module (*e.g.* crop residues used as mulch on cropland)	12
		Diversity	Degree of species richness within a module (*e.g.* crop diversity, diversity of livestock)	37
	Quantification methods	Mechanistic modelling	Mechanistic or functional representation of the system elements, their mutual interactions and with the environment	42
		Statistical modelling	Statistical laws and assumptions to generate sample data	44
		Index construction	Data aggregation to build a variable index	20
		Farmers estimates	Quantification of resilience attributes and farm's performance based on farmers estimations	5
	Climate timeline	Current climate		86
		Current climate vs future climate projections		22
		Future climate projections		3
	Stakeholder engagement	Yes		34
		No		77
Performance-based	Dimensions of performance	Agricultural productivity		64
		Economic profitability		44
		Environmental performance		23
		Social performance		6
	Metrics of performance	Average	Indicator mean or median value	50
		Variability	Stability around an average value	20
		Probability	Cumulative distribution function of the indicator	25
		Recovery time	Time for farm performance to recover from hazard(s) impact	1
		Trend	Slope coefficient of the linear regression over time	1
	Impact on farm performance	Positive		40
		Neutral		2
		Negative		12

For each study of the *performance-based* body of literature, we classified the indicators of farm performance into four dimensions, namely agricultural productivity, economic profitability, environmental performance and social performance. We also recorded five metrics of performance used in the reviewed studies to quantify the indicators: average (or median), variability, probability to fall below or above a given threshold, time to recover from a change or shock and trend over a period of time (Table 1). For instance, a study could quantify the probability for a farm to be food insecure by calculating over a time series the number of years for which the amount of available calories did not meet farm requirements. We then listed the positive and negative impacts of a change in a resilience attribute on the indicators considered in the study. When an increase in a given attribute had a positive (or negative) impact on a number of indicators in a given study, we aggregated this as one occurrence of a positive (or negative) impact of the attribute on farm performance. If a study found two different impacts (*e.g.,* one positive and one negative), we reported both. This ensured that studies that reported the impact of an attribute on several indicators were given equal weight as those that reported the impact on a single indicator. We present the results of our analysis in Sections 3 and 4, sections in which we only refer to the 204 studies from our systematic review.

## Definition and assessment of farm resilience

### Definition of resilience

Across the three bodies of literature, a limited number of studies belonged to the resilience assessment literature, *i.e.,* explicitly claimed to assess resilience (*n*=53). Out of these 53 studies, 41 studies provided a clear definition of resilience (Supplementary Materials, Table S1). Most of the studies defining resilience referred to previous academic literature or reports from international research organizations and nine studies proposed their own definition of resilience. All definitions of resilience in these studies included the response of the system functions (*e.g.,* food production) in the face of an external disturbance (*e.g.,* shock, stress). The dynamics of these functions differed according to the definitions in the studies. Fifty four percent of the studies (*n*=22) stated that a resilient farm is characterized by its ability to sustain its functions over time. This definition does not consider the possibility of a progression of the farm functions over time, thereby excluding the farm developmental trajectory (Figure 4B). For instance, Rodriguez et al. (2011) defined resilience as "the ability of a farm business to absorb disturbances while remaining productive and profitable". In this definition, improvements in farm productivity and profitability were not addressed. Conversely, 17% of the studies (*n*=7) explicitly stated that farm functions should not only be maintained but improved over time. For instance, Poelma et al. (2021), adapting a definition proposed by Tanner et al. (2015), defined resilience as "the capacity of all people across generations to sustain and improve their livelihood opportunities and well-being despite environmental, economic, social and political disturbances". The 12 remaining studies (29%) provided definitions of resilience that were compatible with this dynamic trajectory of farm development without explicitly mentioning this property. Among them was the definition from Diserens et al. (2018), adapting a definition proposed by Choptiany et al. (2015), that we retained for our analysis as spelled out in the introduction: the "ability of a system to recover, reorganize and evolve following external stresses and disturbances".

### Scope and methods to assess farm resilience

The *perception-based* body of literature encompassed 46% of the 204 reviewed studies (*n*=93). It was composed of studies that employed qualitative methods to assess farm resilience. These qualitative assessments were all based on farmers' perceptions. The authors collected farmers' perceptions of climate risks and climate-related impacts through focus group discussion, surveys and interviews. The two other bodies of literature consisted of studies employing quantitative methods to assess farm resilience. The *attribute-based* body of literature was the smallest and was composed of 17% of the reviewed studies (*n*=35). It included studies that quantified resilience attributes, but without quantifying farm performance. Finally, the *performance-based* body of literature encompassed 37% of the reviewed studies (*n*=76) and was composed of studies quantifying both resilience attributes and indicators of farm performance (Figure 4A).

Most of the 53 studies from the resilience literature belonged to the *perception-based* and the *attribute-based* bodies (respectively *n*=23 and *n*=16). Hence, a relatively small share of the resilience literature drew quantitative links between attributes of resilience and farm performance (*n*=14; 18% of the *performance-based* body studies). The *performance-based* body of literature included a higher proportion of studies that were conducted on non-smallholder farms only (*n*=41 – 54%) than on smallholder farms only (*n*=34 – 45%). Studies that assessed the impact of future climate projections on farm performance belonged mostly to the *performance-based body* (*n*=24/25) and concerned non-smallholder farms (*n*=16/25).

Within the *performance-based* body, the studies relied mostly on mechanistic crop, livestock or farm models (55% – *n*=42) or on statistical modelling (41% – *n*=31) to quantify farm performance (Figure 4C). Among the studies using mechanistic modelling, only seven of them used *in situ* measurements to calibrate their model (Bobojonov and Aw-Hassan 2014; Poulton et al. 2016; Mahmood et al. 2017; Hochman et al. 2017; Descheemaeker et al. 2018; Homann-Kee Tui et al. 2021; Garba et al. 2023). Two studies calculated a risk efficient frontier (*i.e.,* the highest return at a given level of risk), one study to optimize crop-livestock integration in dairy farms (Bell et al. 2021) and the other to optimize combinations of crops and trees in agroforestry farms (Paul et al. 2017); both examining farm profitability and financial risk. A relatively small number of studies in the *performance-based* body of literature (*n*=21) involved stakeholders to assess resilience. For instance, Hochman et al. (2017) combined participatory research methods with on-farm experiments and scenario modelling.

### Dimensions and metrics to quantify farm performance

**Fig. 5:**
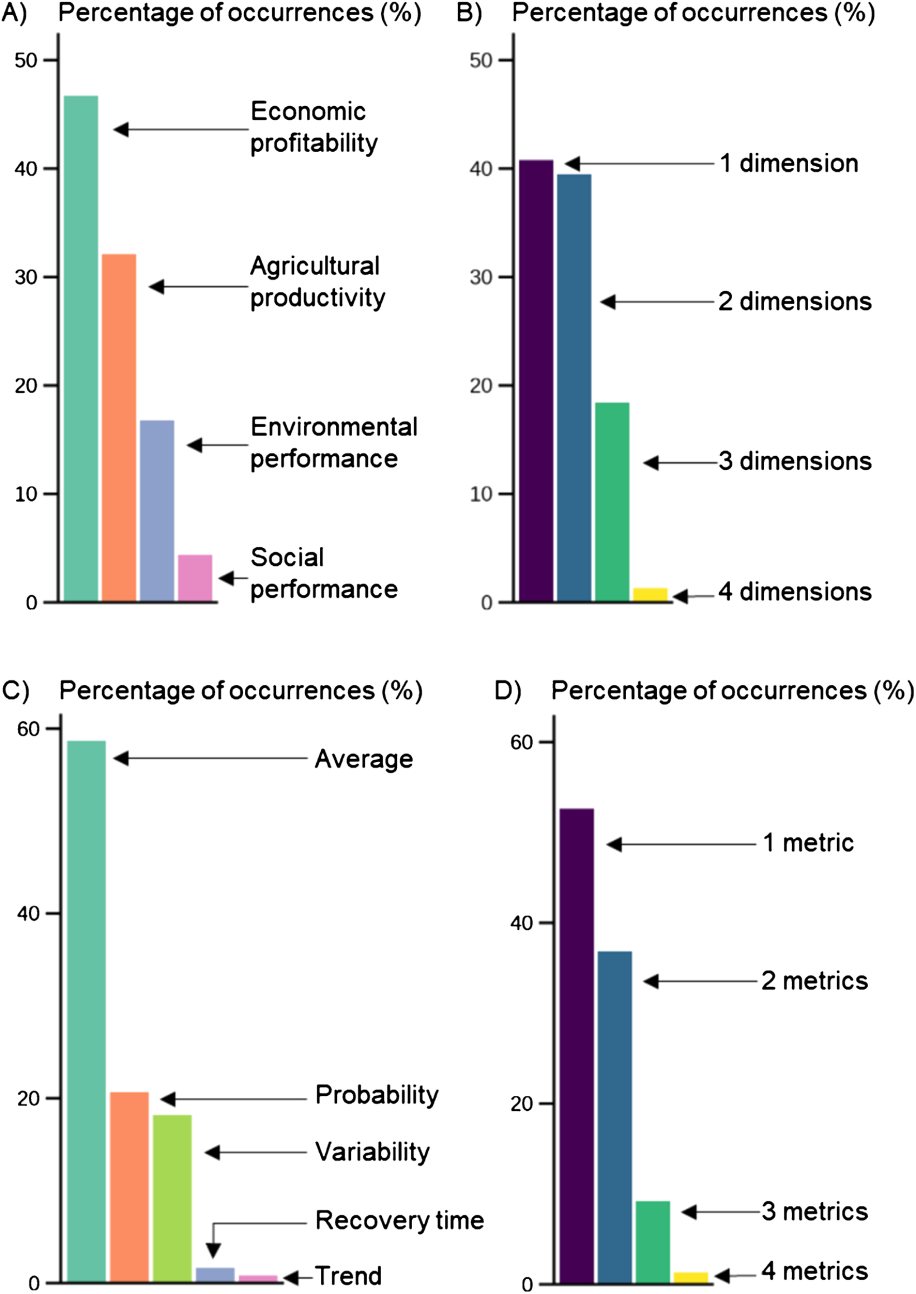
For the performance-based body of literature (*n*=76), distribution of **A**) the occurrence of dimensions of performance; **B**) number of dimensions of performance assessed per study; **C**) the occurrence of metrics of performance used to quantify indicators; **D**) number of metrics of performance used per study

In most of the studies, the farm performance indicators were related to economic profitability or agricultural productivity (Figure 5A). The environmental dimension and the social dimension of performance were the least studied dimensions. Most reviewed studies addressed one or two dimensions of farm performance, while studies addressing three dimensions were less frequent (20%, n=15) and only one study assessed all of the four dimensions (Figure 5B). There were more studies assessing the environmental and social dimensions of performance (n=18) and more studies assessing three or more dimensions of performance (*n*=11) in the non-smallholder context than in the smallholder context (respectively *n*=8 and *n*=4).

A total of 43 different indicators were used to assess performance at farm level (Table 2). Among the 64 studies assessing economic profitability, 59 studies used indicators that quantify farm profitability (*e.g.,* net income, gross margin, rate of return). Eleven of the reviewed studies used indicators from the field of economics to assess financial risks resulting from climate uncertainty. For example, the conditional value at risk (*i.e.,* the average farm gross margin in the lowest 20% of the financial years) was used to estimate i) the risk associated with contrasting configurations of mixed crop-livestock farm systems under varying climatic conditions (Ghahramani and Moore 2016; Ghahramani et al. 2020; Bell et al. 2021), ii) the impact of the adoption of weather insurance on risk reduction (Kath et al. 2018), iii) the risk associated with the adoption of conservation agriculture (Monjardino et al. 2021) and iv) the risk of an increase of livestock stocking rate in the context of a drought (Iglesias et al. 2016). The authors of these studies were able to identify the link between farm system configuration, risk adaptation strategies and income loss for varying climate series. These indicators of financial risk were mostly quantified for non-smallholder farms (8/11 studies). Ten studies assessed farm profitability by giving economic values to production factors, for instance to calculate feeding cost related to the performance of the livestock component of the farm or to assess gains in crop productivity related to irrigation.

**Table 2:** List of the 43 indicators used to assess resilience at farm scale in the systematic review of the literature. Indicator occurrences are in brackets

Farm dimensions	Indicator	Units
Agricultural productivity	Yield (21); Feed production (4); Dietary energy production (2); Annual net primary productivity (1)	Unit of production per unit of surface; Megajoule per farm per year
	Animal production (11)	Unit of production per animal or per unit of surface
	Feed self-sufficiency (5); Dietary energy self-sufficiency (4)	Unit of production or unit of production per time unit or production/energy requirement ratio; Probability to reach self-sufficiency (*e.g.* number of self-sufficient years)
	Animal or crop mortality (4)	Number of years with mortality or mortality rate
	Land equivalent ratio (2)	Score
	Technical efficiency (2)	Score
	Labour productivity (1)	Unit of production per unit of time
Economic profitability	Profit (41); Gross margin (11); Rate of return (9); Net present value (4); Benefit/Cost ratio (1)	Currency or currency per unit of production or per unit of time or per unit of surface
	Production costs (7)	Currency per production or per unit of surface or per year
	Conditional value at risk (6); Certainty equivalent (5); Risk premium (2); Fair premium (1); Mean root square loss (1)	Currency or currency per unit of surface or per unit of time
	Water productivity (3)	Currency per hectare or currency per hectare per millimetre of water
	Poverty score (3)	Score
	Total factor productivity (2)	Score
	Volume of exportation (1)	Livestock unit per year
Environmental performance	Greenhouse gases balance (10)	Unit of reactive per product weight or per surface
	Water use (7); Water use efficiency (7)	Cubic meter or millimetre (per hectare); Kilogram of crop per hectare per millimetre
	Soil erosion (6)	Degree of ground cover or carbon erosion factor or ton of eroded soil per hectare
	Nitrogen footprint (4); Nitrogen use efficiency (3)	Unit of reactive per product weight or per surface (*e.g.* gram of nitrogen per kilogram of milk); Unit of production per unit of nitrogen per unit of surface
	Energy footprint (3); Energy productivity (1); Energy use efficiency (1)	Megajoule, kilowatt per hour or megajoule per kilogram of meat; Unit of production per megajoule; Ratio of energy output to the energy input
	Carbon sequestration (2)	Ton of carbon per hectare
	Ecosystem services (1)	Currency
	Pesticide use (1)	Pesticides load index
	Transpiration efficiency (1)	Transpiration ratio
Social performance	Workload (4)	Unit of time or number of worker per unit of time or unit of time per surface (*e.g.* number of men per day, number of hours per hectare)
	Sociocultural services (1)	Currency
	Number of jobs on farm (1)	Unit
Combination of the four dimensions of performance	Social gross margin (1)	Currency
	Net present value (1)	Currency
	Benefit/Cost ratio (1)	Currency

Among the 44 studies that assessed farm agricultural productivity, 34 studies used indicators related to crop and/or animal production, expressed in dry biomass content or energy value (*e.g.,* grain yield in tons per hectare, metabolizable energy in megajoules per hectare). Some indicators were only used to assess the performance of smallholder farms. Such indicators included farm self-sufficiency for food production (*n*=4), probability of production failure (*n*=4) or farm technical efficiency (*i.e.,* the ability of a farm to attain the highest level of output given a set of inputs, *n*=2). Indicators related to feed production and feed self-sufficiency were mostly used to assess performance of non-smallholder farms (7/9 studies). Two studies calculated a land equivalent ratio to compare land use efficiency between sole cropping and intercropping systems (Paul et al. 2017; Rezgui et al. 2024) and one study measured labor productivity (Mishra et al. 2021).

Farm environmental performance was assessed by 23 studies that quantified the consumption, the efficiency in use and the footprint of natural and synthetic resources. Indicators were related to water (*n*=13), greenhouse gases (*n*=10), nitrogen (*n*=5), energy (*n*=5) and pesticides (*n*=1). Soil health was assessed through erosion (*n*=6) and soil carbon sequestration (*n*=2). Ecosystem services were assessed through crop transpiration efficiency (*n*=1) and using an econometric approach (*i.e.,* choice experiment and contingent valuation, *n*=1) to attribute a non-market value to ecosystem services (*e.g.,* biodiversity, water pollution reduction - Alcon et al. 2024).

Farm social performance was the least assessed dimension of farm performance (*n*=6). Most of the studies quantified workload on farm (*n*=4), one study assessed the number of jobs on farm as an indicator of rural employment (Herrera and Kopainsky 2023) and another one used an econometric approach to attribute a non-market value to sociocultural services (*e.g.,* landscape beauty, cultural heritage - Alcon et al. 2024).

Indicators were quantified through their average values, probability of exceeding a critical threshold, variability, recovery time and trend over a period of time (Figure 5C). Variability of performance in smallholder farms was assessed in a relatively small number of studies (6/22 studies). The time to recover from a shock, which is an acknowledged indicator of resilience in the literature, was only used in two studies, in smallholder (Williams et al. 2020) and non-smallholder farms (Herrera and Kopainsky 2023). Four studies quantified the trajectory of indicators of farm performance over a period of time. One study used the trajectory itself as a metric by calculating its trend (*i.e.,* the slope coefficient of the linear regression of the trajectory - Dardonville et al. 2022) while the three other studies described qualitatively the curve of the trajectory. Forty seven percent of the studies used only the average value metric to assess farm performance. The majority of studies relied on one or two indicator metrics while 11% of the studies combined three metrics or more (*n*=8) (Figure 5D).

### Attributes of farm resilience

**Fig. 6:**
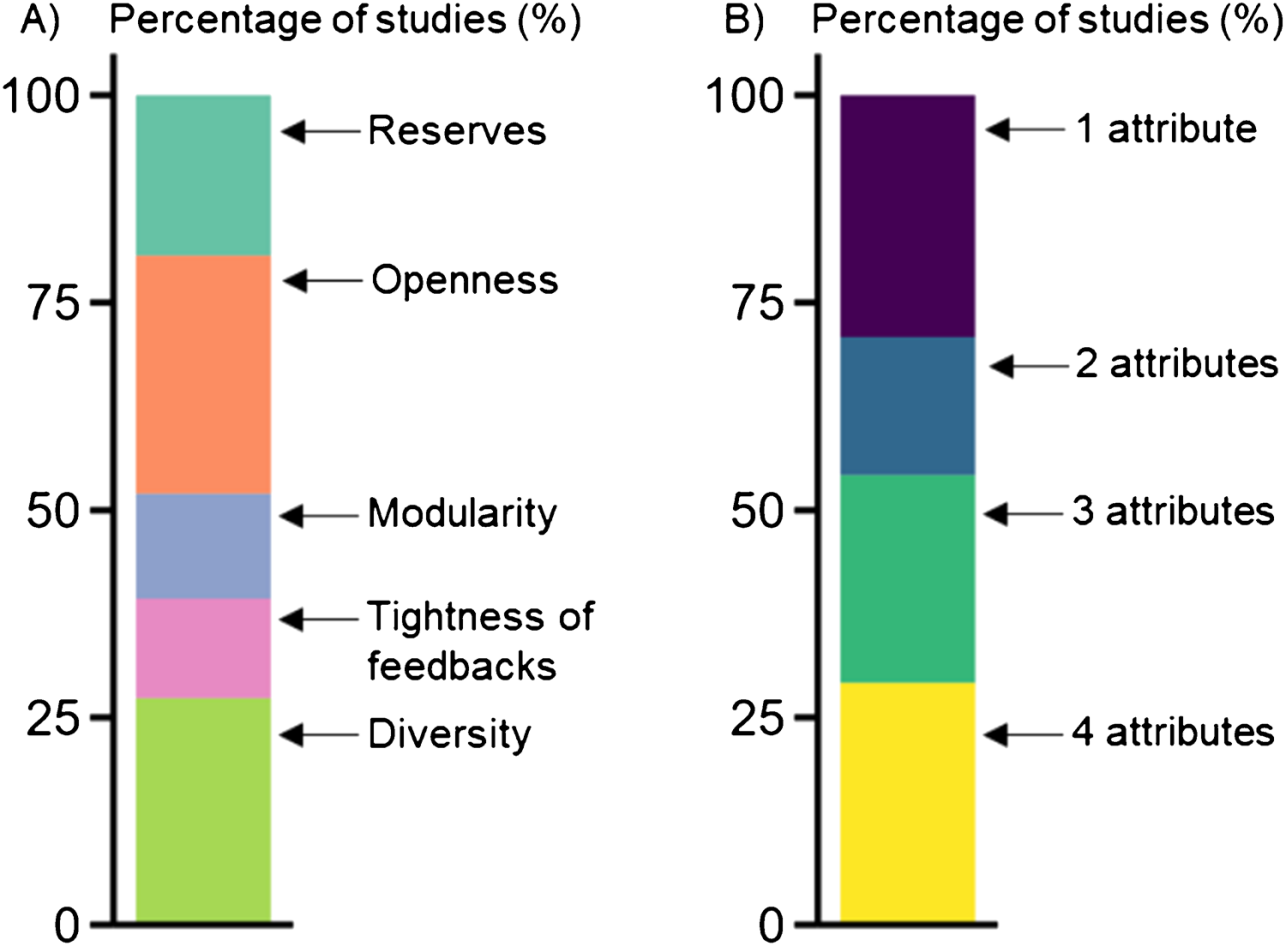
For the performance-based body of literature, distribution of **A**) the occurrence of attributes of resilience; **B**) number of attributes of resilience quantified per study

Within the *performance-based* body of literature, most studies assessed the impact of the three following attributes on farm performance: openness (29% of the studies, *n*=43), diversity (27%, *n*=41) and reserves (19%, n=29). Tightness of feedbacks and modularity were less often assessed, in 12% and 13% of the studies (*n*=18 and *n*=19), respectively (Figure 6A). The majority of studies assessed the impact of one or two attributes on farm performance and none of the studies included all five attributes (Figure 6B). There was no difference between studies assessing smallholder and non-smallholder farms regarding the share of attributes assessed, except the diversity attribute which was assessed more often in non-smallholder farms (60% of the studies, *n*=24), or regarding the number of attributes assessed per study. In what follows we describe what these different attributes entailed at farm level.

#### Reserves

The abundance of a given resource was used to assess the impact of farm reserves on farm performance. Among the 29 studies assessing the reserves attribute, 26 studies assessed the impact of natural resources (*e.g.,* livestock, land, soil fertility). Five studies assessed the impact of labor resources (*i.e.,* number of available workers) (*e.g.,* Souissi et al. 2018), six studies accounted for various farm resources to calculate the overall farm resource endowment (only for smallholder farms, *e.g.,* Rigolot et al. 2017) and one study quantified the number of mechanized equipment (Lopez-Ridaura et al. 2018).

#### Openness

The openness attribute encompassed all the interactions between the farm system and the socio-economic and ecological environment outside the farm. These interactions included flows of inputs (*e.g.,* mineral fertilizer, hybrid seeds, *n*=23) (*e.g.,* Kadigi et al. 2020), access to infrastructures (*e.g.,* access to irrigation, *n*=18) (*e.g.,* Hochman et al. 2017) and access to services from the public and the private sector (*e.g.,* access to insurance or extension services, volume of subsidies, *n*=10) (*e.g.,* Lehmann et al. 2013). Access to credit, insurance, production contract or to subsidies was only assessed for non-smallholder farms.

#### Modularity

Farm modularity was measured through the number and the type of farm modules (see Figure 3) in 19 studies. These studies assessed the relationship between the number of farm modules and farm performance. The combination of the cropping and livestock modules was dominant (*n*=9, *e.g.,* Bell et al. 2021), followed by the off-farm activity module (*n*=5, only for smallholder farms, *e.g.,* Williams et al. 2020). The rest of the studies described the number of production and income generating activities (*n*=5, *e.g.,* Rufino et al. 2013) and the integration of a forestry production module on the farm (*n*=1, Djanibekov and Khamzina 2016).

#### Tightness of feedbacks

Tightness of feedbacks was measured through the extent of flows between and within farm modules in 18 studies. The amount of feed produced by the cropping system module going to the livestock module was the most frequently explored feedback (78% of the studies, *n*=14) (*e.g.,* Lurette et al. 2013). The feed included fodder from pasture, crop residues or grains. The other cases related to tightness of feedbacks were other uses of crop residues (only for smallholder farms), mainly as mulch for soil cover (*n*=4, *e.g.,* Rigolot et al. 2017), or uses of manure, mainly as organic fertilizer (*n*=4, *e.g.,* Descheemaeker et al. 2018).

#### Diversity

Farm diversity was mostly investigated within the cropping system module and less frequently within the livestock module. The number of crop species cultivated on the farm was used by most of the studies to measure diversity, ranging from two to more than ten crop species with an average of seven crop species per study (n=36). Nineteen studies assessed the impact of legume diversification (integration of one to eight legume species) on farm performance. One study investigated crop diversification through the combination of several crops on the same field by comparing sole maize with different combination patterns of maize and teak (*e.g.,* alley cropping) (Paul et al. 2017). Three studies measured the effects of the number of plant species in pastures on the productivity and profitability of fodder production (Martin and Magne 2015; Duranton and Matthew 2018; Monjardino et al. 2022). Finally, three studies directly linked farm performance with the diversity in livestock species and races (Toni and Holanda 2008; Millar et al. 2009; Castaño-Sánchez et al. 2023).

## Impact of resilience attributes on farm performance

**Fig. 7:**
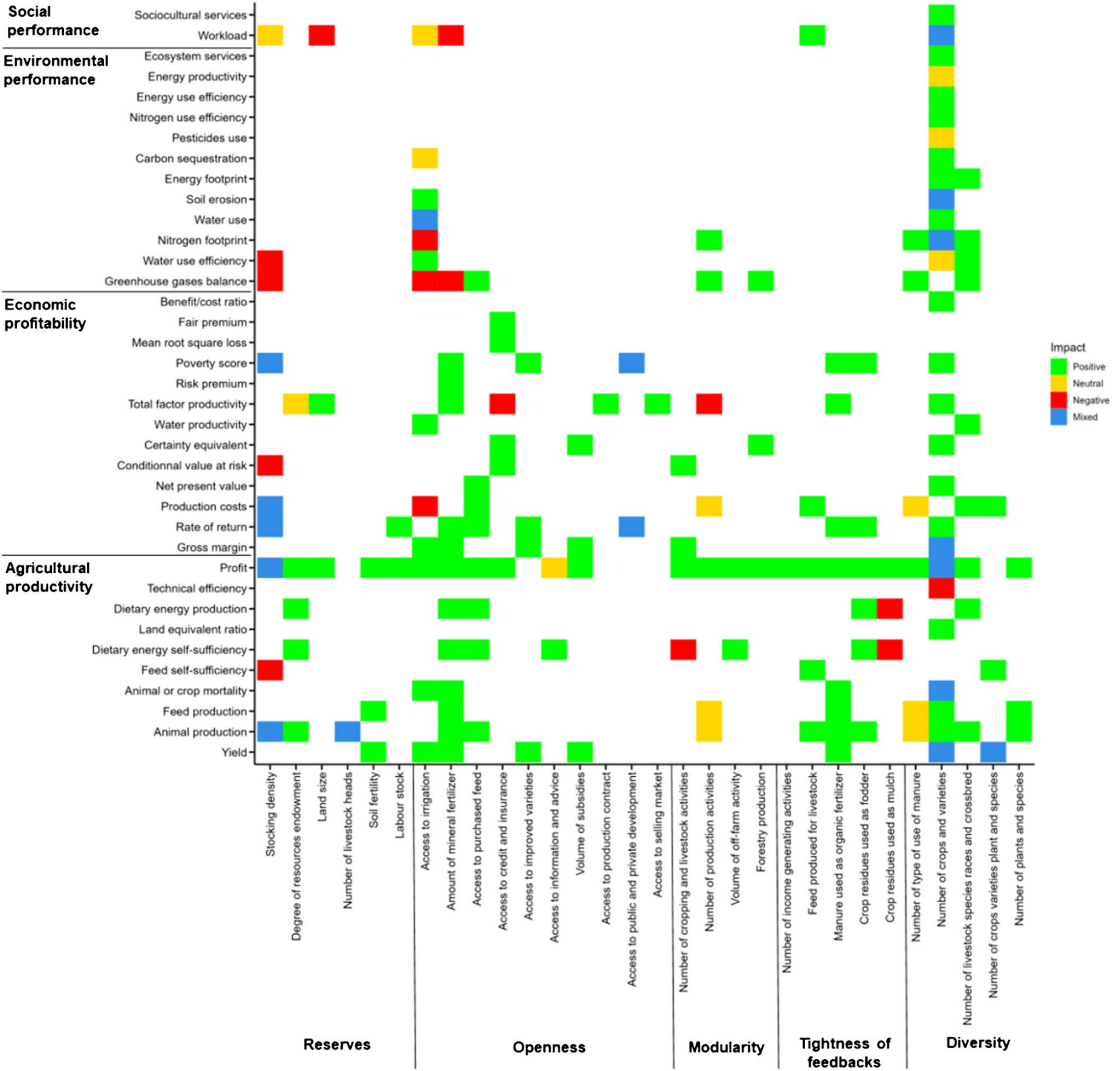
Impact of a given attribute of resilience on a given indicator of farm performance. Colours discriminate the type of impact. Mixed impacts are represented when at least two studies showed opposite impacts (*i.e.,* at least one positive and one negative impact) and when the same study showed opposite impacts

Within the 76 studies of the *performance-based* body, 42 studies assessed the impact a single attribute of resilience on a given indicator of farm performance for a given metric (Figure 7). Forty one studies assessed the impact of several attributes at a time on a indicator of farm performance (including 26 studies from the 42 mentioned above). In the sections below, we give examples of: (i) the links (positive or negative) between a single attribute and farm performance for each of the five attributes of resilience, and (ii) the links between a combination of attributes and farm performance.

### Relation between a single resilience attribute and farm performance

**Fig. 8:**
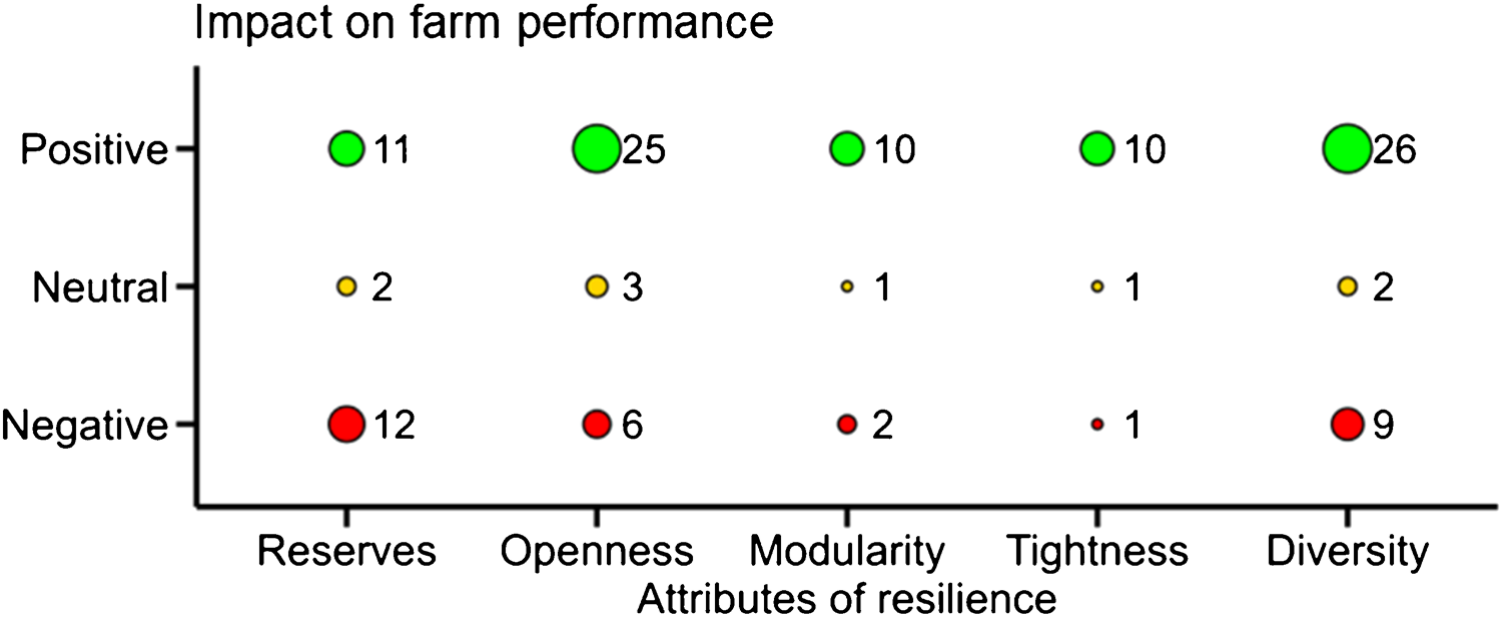
Impact of resilience attributes on farm performance from studies of the systematic review assessing the impact of one attribute on one performance indicator. Dots and numbers are numbers of studies

Out of the 42 studies drawing a direct link between an increase of a resilience attribute and farm performance, 17 studies found one type of impact only (*e.g.,* a positive effect), while 25 studies found contrasted impacts of resilience attributes on farm performance (*e.g.,* one positive and one negative effect). In total, 40 studies found a positive effect (*i.e.,* an increase of farm performance), 24 studies found a negative effect (*i.e.,* a decrease of farm performance) and seven studies did not conclude on any effect (*i.e.,* no significant change of farm performance) of an increase in resilience attribute (Figure 8).

An increase in the number of cultivated crops (*diversity*) was both increasing and stabilizing farm productivity (Lehmann et al. 2013). An increase in off-farm activity (*modularity*) increased the probability to be food secure after a drought and decreased the time to recover from this shock (Williams et al. 2020). The establishment of a forestry production module in the farm was increasing total farm carbon sequestration with increased probability of high return to investment (Djanibekov and Khamzina 2016). Access to crop insurance (*openness*) increased the performance of non-smallholder farms. For example, Kath et al. (2018) showed that weather insurance for sugarcane farmers in Australia decreased farm economic downside risk and increased farm profitability. Higher resources endowment (*reserves*) guaranteed both the increase and stabilization of food security and household income for smallholder farmers in semi-arid Zimbabwe under projected future climatic conditions (Descheemaeker et al. 2018; Homann-Kee Tui et al. 2021). Increasing the feedbacks between cropping and livestock activities, *e.g.,* with the use of manure as an organic amendment or the use of crop residues to feed the animals (*tightness of feedbacks*) increased average agricultural production and farm economic profitability (Homann-Kee Tui et al. 2021). The establishment of a manure-based biogas production component in dairy farms (*tightness of feedbacks and modularity*) decreased the carbon and reactive nitrogen footprints with a positive impact on farm profitability (Rotz et al. 2016). Crop-livestock integration (*modularity* and/or *tightness of feedbacks*) was investigated for its impact on farm performance in 15 studies. For all of these studies (*n*=15), crop-livestock integration increased farm performance by decreasing farm financial downside risk, increasing average farm productivity, profitability and environmental performance and stabilizing farm productivity and profitability. For instance, for non-smallholder farms in Australia, Bell et al. (2021) showed that the livestock component of a farm increased the stability of productivity and profitability of the cropping component. All resilience attributes did not have the same magnitude of performance impact, some attributes being more effective than others in increasing farm performance. As an example, Rigolot et al. (2017) showed for a smallholder farm context in Burkina Faso, that intensification with mineral fertilizer (*openness*) was much more impactful in increasing crop production than the use of crop residues as mulch (*tightness of feedbacks*). This study also highlighted the trade-offs related to this intensification; under current and future climate conditions, the probability of negative income was higher for the scenarios using mineral fertilizer than for those using crop residues as mulch.

Among the negative impacts of a resilience attribute on farm performance, the increase in the number of crops (*diversity*) resulted in a higher frequency of crop failure and lower average profitability when too many of the crop cycles exceeded the rainfall season length and hampered appropriate management (Dalgliesh et al. 2016; Carauta et al. 2021). Poor choices of crops and crop rotations also decreased farm environmental performance by increasing nitrogen leaching (Mahmood et al. 2017). An increase in livestock stocking density (*reserves*) was always found to increase farm average productivity and profitability for both smallholder and non-smallholder farms under favorable climatic conditions, but it was riskier due to increased sensitivity to climate hazards, especially droughts that reduced fodder production (Millar et al. 2009; Scott et al. 2013; Phelan et al. 2015; Iglesias et al. 2016; Descheemaeker et al. 2018; Bilotto et al. 2019, 2021; Homann-Kee Tui et al. 2021). Higher stocking densities were associated with larger feed requirements and this increased the probability of feed shortage and the variability in farm income. Higher stocking densities were also related to an increase in greenhouse gas emissions (Bilotto et al. 2019), thus decreasing farm environmental performance.

### Relation between combined resilience attributes and farm performance

A contrast was observed in how authors combined attributes to assess farm performance between studies dealing with non-smallholder (*n*=21) and studies dealing with smallholder farms (*n*=20). Most of the studies dealing with non-smallholder farms were livestock-oriented or mixed crop-livestock systems (57%, *n*=12). To improve the performance of livestock production, such studies assessed two or more attributes by combining for instance livestock and cropping activities (*modularity)* to increase and sometimes to stabilize farm profitability, by increasing livestock stocking density (*reserves*), feed production (*tightness of feedbacks*), feed purchase (*openness*) or crop diversity to enhance feed quality and quantity (*diversity*) (Martin and Magne 2015; Pinsard et al. 2023). The studies dealing with smallholder farms combined more resilience attributes than the studies dealing with non-smallholder farms. In such studies, the impact of attributes related to climate smart practices (*e.g.,* legume diversification) and production intensification (*e.g.,* use of mineral fertilizer, irrigation) was tested with the ambition to improve food security and farm income of smallholder systems (*e.g.,* Rigolot et al. 2017; Lopez-Ridaura et al. 2018). These studies showed that such scenario strengthened the development of smallholder farms but also increased the sensitivity of farm performance to climate-related risks, even if performance variability was not often investigated. In the studies dealing with smallholder mixed crop-livestock systems, *openness* attributes were combined with *tightness of feedbacks* attributes to optimize interactions between farm components (*e.g.,* use of manure to fertilize crops and use crop residues to feed animals), but also with *crop diversification* and various *reserves attributes* (*e.g.,* number of cattle heads, land size, number of available workers, soil fertility).

Several studies evidenced interactions between resilience attributes, for example when increasing two attributes at a time did not necessarily lead to better farm performance while improvements in single attributes did. The use of a production contract (*openness*) was a risk-decreasing solution to support farm productivity (Vigani and Kathage 2019). However, combined with other attributes (*e.g.,* through an increase of the number of activities, *modularity*, or an increase of the number of crops, *diversity*), it decreased farm productivity, especially for farms with high exposure to natural hazards. The study assessed the combination of one to four different attributes and concluded that targeting several resilience attributes resulted in a complex management for the farmer and was associated with the largest losses in farm productivity. In another study, Komarek et al. (2012) showed that when crops and livestock were combined (*modularity*), increasing the number of cattle heads (*reserves*) was increasing farm profitability but decreasing farm food self-sufficiency.

### Studies that assessed multiple dimensions and metrics of farm performance

Of the eight studies from the *performance-based* body of literature that combined more than two metrics of performance, four studies showed that as the average value for a specific indicator of farm performance increased, both the variability and the likelihood of the indicator to exceed a critical threshold decreased. Conversely, the four other studies showed that as the average value for a specific farm performance indicator increased, the stability and the probability to stay within a range of desired values for one or several indicators of performance decreased, threatening farm resilience on the longer term.

Out of the 15 studies that assessed more than two dimensions of performance, five studies showed an increase in all dimensions of performance. For instance, increasing agrobiodiversity on smallholder farms in Uganda (Kozicka et al. 2020) and implementing agroforestry of maize and teak on non-smallholder farms in Panama (Paul et al. 2017) were increasing farm productivity and profitability, and stabilizing profitability, whilst decreasing soil erosion and increasing soil carbon sequestration. On the other hand, six studies showed that an increase of the agricultural productivity and/or the economic profitability was reached at the cost of the environmental and/or the social performance. For example, Rezgui et al. (2024) showed that the implementation of alley cropping in non-smallholder farms in Italy increased and stabilized farm profitability both in the short and the long term but at the cost of a higher workload. The four remaining studies showed contrasted results depending on the climatic conditions and the combination of attributes, like Ghahramani and Moore (2016) who found that under future climatic conditions in Australia, the combination of four resilience attributes (*i.e., openness*, *diversity*, *modularity*, *reserves*) was not enhancing any of the three dimensions of non-smallholder farm performance, except for the sites with high rainfall, highlighting the climatic risk for farms located in dry areas.

The impact of drought on average livestock product exports (economic profitability) and on-farm jobs creation (social performance) and their related recovery times were simulated to assess resilience of livestock-oriented farms in France. These metrics were used to explain the trajectories of these two indicators for different scenarios of land availability and levels of market integration (Herrera and Kopainsky 2023). In another study, the trend of eight years of farm gross margin was computed in combination with two other dimensions (agricultural productivity and social performance) and three other metrics of performance (average, variability and probability to exceed a threshold) (Dardonville et al. 2022). Farms with a higher level of ecosystem services had more stable productivity and profitability with lower workload (*i.e.,* higher social performance) than input-based intensified farms that were more productive, profitable and resistant to shocks but less stable. These recent examples of comprehensive resilience assessments quantified farm trajectories and highlighted the inherent trade-offs between dimensions and dynamics of farm performance.

### A lack of comprehensive assessments of the impact of resilience attributes on farm performance

Our analysis indicates that, in many cases, an increase in the value of resilience attributes (as defined by Meuwissen et al. 2019) leads to greater farm performance. In other words, the often "assumed" relationship between resilience attributes and farm performance seems to hold (see section 4). The options to increase farm resilience attributes are broad and diverse: cultivating a larger number of crops/cultivars or raising a larger number of livestock species and breeds (*diversity*), including an off-farm activity (*modularity*), adopting a production contract or importing feed, mineral fertilizer or irrigation water (*openness*), increasing labor availability or enlarging herd size (*reserve*), increasing on-farm fodder and manure production (*tightness of feedbacks*). A limited number of studies from the literature of climate risk and vulnerability assessment (*n*=17/21) and from the literature of resilience assessment (*n*=4/21) combined multiple dimensions and metrics of performance over space and time to explicit the dynamics and the trade-offs that resulted from the impact of attributes of resilience on farm performance. With this type of comprehensive assessment it was possible to draw quantitative links between resilience attributes and farm resilience and to identify the conditions under which this link would be positive or negative.

However, the assessment of farm performance and its dependence on resilience attributes was only partial in most of the reviewed studies, *i.e.,* relying only on one dimension of farm performance (*e.g.,* agricultural productivity) and relying only on the average as the metric of the performance, *i.e.,* excluding the other metrics of performance. For example, a farm could perform overall well (with regard to agricultural productivity) but very poorly with regard to the environment, thus jeopardizing its long-term functioning (*e.g.,* if it depletes soil and water reserves). Similarly, the average performance can hide strong variations including very low performance during particular years (*e.g.,* dry years) that might be unaffordable for smallholder farms. Yet, this comprehensive understanding of resilience is critical for smallholder systems that are characterized by both a high vulnerability to climate-related hazards and a strong need to improve farm performance.

## Towards a comprehensive assessment of farm resilience tailored to smallholder farms

### Recommendations for quantitative assessment of farm resilience based on farm performance

In the previous sections we critically analyzed how the literature assessed the impact of resilience-enhancing attributes on various dimensions and metrics of farm performance and to which extent these assessments helped to conclude on farm resilience. From this analysis, we derive five recommendations to improve the analysis of farm resilience against climate-related risks, with particular attention to the context of smallholder farms.

First, a comprehensive assessment of farm resilience encompasses the four dimensions of farm performance. Agricultural productivity and economic profitability were assessed in most of the reviewed quantitative studies with a large diversity of relevant indicators. The environmental and the social dimensions were seldom explored. Yet, the farm depends on surrounding natural resources (*e.g.,* soils, water) and on social welfare (*e.g.,* acceptable workload) to maintain its long-term functioning. A thorough environmental assessment, for example including soil erosion (*e.g.,* Kozicka et al. 2020) or water and nitrogen use efficiency (*e.g.,* Mishra et al. 2021), is critical to understand the sustainability of the use of natural resources by the farm. Surprisingly, only two studies assessed soil health through carbon sequestration and water contamination with agrochemicals was not considered in the reviewed studies. Yet, other studies in the literature (not included in the results of our query) included indicators related to soil health and the use of agrochemicals in their assessment of climate-related risk impact on farm environmental performance. For instance, in one of such study soil health was addressed through assessing the impact of crop-livestock integration (thus considering the attribute *tightness of feedbacks*) on nitrogen and phosphorus stocks and flows as a result of crop diversification (*diversification*) and grazing management in Brazil (Szymczak et al. 2020). Another such example is Pacini et al. (2003) who compared farm environmental performance of different farm managements in Italy using an extensive set of indicators related to soil nutrition and erosion, pesticides and biodiversity. Rusinamhodzi et al. (2012) quantified the social acceptability of maize-legume intercropping in Mozambique based on a score given by the farmers to evaluate its contribution to food security and income, the reduction of input costs and the facilitation of mechanical weeding. Most of smallholder farms currently have a much larger potential to improve their agricultural productivity and economic profitability due to a much larger yield gap than large-scale commercial farms (Dzanku et al. 2015; Silva et al. 2021). For such improvement to happen, the use of external inputs (*e.g.,* fertilizer, pesticides) and the adoption of climate-smart practices (*e.g.,* intercropping cereals and legumes) might be required in order to intensify the production and adapt farms to climate change. A comprehensive understanding of resilience is therefore critical for a development of smallholder farms that would follow a pathway avoiding the negative environmental impact associated with intensive farming as observed in high-income countries (*e.g.,* Stoate et al. 2001) and complying with criteria for social acceptance.

Second, farm resilience assessment encompassing the dynamics of farm performance in the face of climate-related hazards associates metrics of average performance with metrics capturing variations of performance. Commonly used metrics of that kind are the variability of the performance, the probability of the performance to be above or below a critical threshold and the time for farm performance to recover after a shock. In the reviewed literature, critical thresholds were defined based on statistical approaches (*e.g.,* lower or upper quartile as in Shrestha et al. 2020), on stakeholder expertise (*e.g.,* degree of crop yield variation compared to a baseline as in Poulton et al. 2016), and on widely acknowledged thresholds defined by international organizations (*e.g.,* poverty line, as in Mwinuka et al. 2016). The relevance and difficulty to define thresholds for environmental performance was highlighted by Pacini et al. (2003) (not included in the results of our query). This study showed that the environmental performance of different farms was strongly influenced by their differences in pedo-climatic conditions, and that the application of the same threshold to all the farms was not relevant. However, concrete guidelines and harmonized workflows on how to set thresholds are currently missing. In complement to combining metrics of performance, analyzing the performance trajectory in multiple dimensions over time allows to explicit trade-offs between short- and long-term performance.

Third, careful trade-off analysis at farm level can guide the redesign towards more resilience. Indeed, our analysis of the literature showed that inappropriate redesign towards increased diversity (*e.g.,* too many crops with labor requirements exceeding the labor force available or too much livestock with feed requirements exceeding the available feed resources of the farm, (*e.g.,* Dalgliesh et al. 2016; Phelan et al. 2015) can lead to lower average farm performance and/or greater risk of poor performance, hence lower farm resilience. Solely aiming at increasing the value of a specific resilience attribute can lead to undesirable outcomes. Smallholder farmers are managing complex systems combining a high diversity of crops and livestock with off-farm activities. This management is conducted in constraining bio-physical and socio-economic environments which lead farmers to compromise with many trade-offs (Morton 2007; Tittonell and Giller 2013).

Fourth, farmers’ involvement is critical to identify and discuss adoption barriers to climate-resilient practices and the design of future climate-resilient farm systems (Rivington et al. 2007), especially for low-resource smallholders who are likely to be risk averse (*e.g.,* Kisaka-Lwayo and Obi 2012). Farmers can help to describe and understand the relations between the farm and its biophysical and socio-technical environment. For instance, farmers in the Himalayas use a short sowing window, even though a large sowing windows is a more common strategy to spread the climate risk across the rainy season (Aase and Vetaas 2007 - not included in the results of our query). The authors showed that this practice, that could be considered as “non resilient” at first sight, stemmed from collective work organization that operated as “social glue” for the community identity. This result highlights the importance of the socio-cultural context. Combining farmers’ perspectives with those of other key stakeholders (*e.g.,* policymakers, extension services) is critical to identify the opportunities and constraints that are needed to guide the redesign of farm systems and to formulate the scenario and policy recommendations that underpin the farm redesign (Souissi et al. 2018; Nera et al. 2020; Cradock-Henry 2021). The smallholder context is characterized by a diversity of environments (*e.g.,* agro-ecological, socio-cultural) and farms that need to be taken into account to tailor suitable climate-resilience practices (Giller et al. 2015). Iteration loops that allow scenario adjustments thanks to famers' participation in scenario assessments, as described by the DEED framework (Describe, Explain, Explore and Design), can help design resilient farm systems that account for farmers' practical challenges (Descheemaeker et al. 2019). We believe that this type of integrated assessment of farm resilience is a relevant method to tackle both the complexity of smallholder systems and the systemic dimension of resilience.

Lastly, some options of crop and livestock management do not fit within the five-attributes framework that was applied in this study (Figure 3). These options include changes in planting dates or crop spatial arrangements (*e.g.,* intercropping) and modifications of feeding scenarios (*e.g.,* Bilotto et al. 2019; Mutenje et al. 2019). Yet the literature shows that they can be useful for resilience building. Optimizing planting date helped to minimize the risks of crop failure of inefficient use of mineral fertilizer (Silva et al. 2019). Intercropping a legume with a cereal gave greater productivity than having the two sole crops (Traore et al. 2023). A livestock feeding strategy with an intermediate backgrounding stage to increase calves weight before placing them in a feedlot was overall more profitable and emitted less greenhouse gases than a strategy directly placing calves in a feedlot after weaning (Bilotto et al. 2019). High flexibility in management was related to low vulnerability when assessing the economic and social performance of multiple specialized and mixed crop-livestock farms over 14 years in France (Sneessens et al. 2019 - not included in the results of our query). Assessing the impact of these options of crop and livestock management would give more weight to farmers’ tactical decisions to cope with climatic hazards and how it impacts farm performance. This can be helpful to identify bottlenecks that prevent the adoption of resilient practices in the context of smallholder farms. Hence, further assessment of farm resilience would benefit from adding a sixth "tactical flexibility" attribute of resilience to the Meuwissen et al. (2019) framework.

### Mechanistic models to assess farm resilience

Mechanistic crop, livestock and farm models can help with the assessment of farm resilience, especially because they allow to simulate biophysical and bioeconomic dynamics (*e.g.,* Ricome et al. 2017; Traore et al. 2023). To be relevant and accurate, these models need to be calibrated against reliable observations. In high-income countries, the models can usually be calibrated against reliable historical series of observed variables. For example, Lehmann et al. (2013) and Shrestha et al. (2020) used series of national yield records and national farm surveys to calibrate the farm models of their studies. However, in low- and middle-income countries and for smallholder farm systems in particular, such detailed panel data do often not exist, are hard to access, or are not reliable neither accurate (Giller et al. 2021). As a result, few of the reviewed studies in smallholder context used mechanistic crop, livestock or farm models (8%) and even fewer calibrated their model(s) against field observations (5%). In most cases, calibration was done against field observations of crop growth variables for the crop models, against literature data (*e.g.,* breed characteristics) for the livestock models and against household survey data for the farm models. Crop models were usually validated using independent observational datasets, while validation of farm models was done through participatory appraisal. A large share of the studies (83%) relied partially or totally on farmers’ estimates or literature values for model calibration. This leads potentially to greater uncertainty in the assessment of crop, livestock and farm performance in relation to climate interannual variability compared with models calibrated with large and qualitative datasets. Compiling and making easily accessible quality data at field (*e.g.,* CGIAR 2023) and household level (*e.g.,* van Wijk et al. 2020) is critical to improve the ability of the research community to robustly calibrate models and assess farm resilience to climate-related risk in the context of smallholder farms. Bioeconomic farm models present the advantage to identify farm management constraints that preclude certain farm or crop management options, *e.g.,* labor availability constraining the adoption of soil mulching (*tightness of feedback*) (Affholder et al. 2010), or constraints related to cash flows at the start of the season limiting the amount of fertilizer that can be bought and applied (Alary et al. 2016). These models can account for smallholders’ objectives and for instance estimate the share of cereals to be planted in the cropland to achieve food self-sufficiency (Gérard et al. 2020). Ultimately, these bioeconomic models also help to identify the policy interventions and institutional arrangements that would favor sustainable crop intensification by alleviating land, labor or cash constraints.

### Simply bouncing back is not an option for smallholder farms

The time required for a farm to recover after a shock was quantified in only two of the reviewed studies (Williams et al. 2020; Herrera and Kopainsky 2023). Examples from other studies in the literature (not included in the results of our query) showed that assessing recovery time can help to estimate the impact of a disturbance and evaluate the relevancy of risk management strategies. For instance in sub-Saharan Africa, statistical modelling of herd demography showed that selling cattle in response to a drought extended herd recovery time and could permanently reduce the herd size (Lesnoff et al. 2012; Aragie and Thurlow 2022). Sabatier et al. (2017) quantified the resistance of Mongolian pastoral systems to extreme events (*i.e.,* maintaining food self-sufficiency and income) and their ability to recover (*i.e.,* reconstituting the herd) based on herd size, herd composition and fodder availability. They highlighted that the calculation of recovery time could help to determine the minimum time during which the farmers would need institutional support to cope with a climate shock. These two examples assessed the ability of livestock systems to cope with a disturbance and to bounce back to a pre-disturbance state.

For smallholder farms, and in sub-Sahara Africa in particular, bouncing back to current low agricultural and economic performance is not desirable (Tittonell and Giller 2013), undermining the relevance of the "time to recovery" metric, at least when applied to farm economic performance. Indeed, increasing agricultural productivity and economic performance are recognized as key strategies to improve food security (*e.g.,* Falconnier et al. 2023b) and reduce poverty (Gérard et al. 2020) in smallholder systems. Therefore, in the case of a farm engaged on such a development pathway, it is essential that it rejoins the upward pathway after a disturbance. In other terms, instead of the recovery of the performance of a farm, one should consider the recovery of the expected growth rate in key performance dimensions (such as food availability and income) of farms following a development pathway. This idea is not particularly new and is advocated by authors such as Barrett and Constas (2014), Dixon and Stringer (2015) and Chaigneau et al. (2022). However, we did not find any applied example of this consideration in resilience assessments of smallholder farms and we believe it is a significant gap in the literature.

## Conclusion

Our study brought evidence from the literature on the impact of resilience attributes on farm performance and farm resilience to climate-related risks, with a focus on the quantitative methods used to assess this impact and the implications for resilience assessment of smallholder farms coping with climate-related risk in low- and middle-income countries. The literature shows that greater attribute values, *e.g.,* more diversity, tighter feedbacks between the crop and livestock components of a farm, generally led to greater farm performance. However, care should be taken on how farm performance is assessed. A narrow focus on a single dimension of farm performance, *e.g.,* agricultural productivity, and an average single value for it, precludes to robustly conclude on the links between resilience attributes and farm performance. Based on our analysis of the current literature, we advocate for more comprehensive studies, that follow six guidelines. The first five of them may apply to farms in general, and are i) the systematic integration of the environmental and the social dimensions when evaluating farm performance, ii) a careful consideration of the variations of farm performance in the face of climate variability, using relevant metrics, iii) a thorough examination of farm-level constraints and institutional levers required to design more resilient farm systems, iv) the incorporation of all types of stakeholder perspectives when defining these constraints and levers, and v) a specific attention to farmers’ management practices and tactical decisions that can improve resilience. Our sixth recommendation is more specific to smallholder farms of low- and middle-income countries and is to consider the development dynamics of the farms. For these farms, the focus is not on their ability to get back to the same status as before a disturbance – a status which involves ongoing poverty and hunger – but rather on their ability to "bounce back better".

## Supplementary Information

Below is the link to the electronic supplementary material.

Supplementary file1 (DOCX 362 KB)

## Data Availability

The datasets generated during and/or analyzed during the current study are available from the corresponding author on reasonable request.
