# Supplementary material for: Farm resilience to climatic risk. A review
Source: Agron Sustain Dev. 2025 Feb 6;45(1):10. doi: 10.1007/s13593-024-00998-w (PMC11802605; doi:10.1007/s13593-024-00998-w)
Supplement: Supplementary file 1 — Supplementary file1 (DOCX 362 KB) [file 13593_2024_998_MOESM1_ESM.docx]

**Supplementary Materials**

### Table S1: Resilience definitions from the 53 studies explicitly referring to resilience assessment and conceptualization of resilience assessment according to the authors. Quoted citations are directly extracted from the text.

| **Study** | **Definition** | **References** |
| --- | --- | --- |
| Adhikari et al., 2023 | No definition | No definition |
| Asrari et al., 2022 | Extent to which a system is able to absorb changes and continue on its path, to cope with external tensions and disruptions resulting from social, political, and environmental changes. Ability of people to adapt and acquire psychological, social, and physical resources to ultimately maintain their health and well-being | Holling, 1973; Adger, 2000; Ungar 2011 |
| Bardsley et al., 2018 | "the capacity of a system to absorb disturbance and reorganize while undergoing change so as to still retain essentially the same function, structure, identity, and feedbacks." | Walker et al. 2004 |
| Ben Nasr et al., 2021 | "individual’s ability to cope with a difficult or stressful situation" |  |
| Cetinkaya Ciftcioglu, 2022 | "Resilience means the capacity of a social-ecological system (e.g. agricultural landscapes), which changes while maintaining the key ecosystem functions and processes, and supporting human livelihoods and wellbeing" [...] "Within this context, the resilience of the farming system means the capacity of the system to ensure the provision of the system functions in the face of complex challenges" |  |
| Chaudhary et al., 2022 | "Robustness of the system that can reduce climatic stresses and impacts, as well as dealing with future risks" | Adger, 2006; Folke, 2006; Speranza, 2010; IPCC, 2014 |
| Collins et al., 2023 | No definition | No definition |
| Cradock-Henry, 2021 | Combination of socio-ecological resilience and disaster resilience concepts. "examines the interrelationships between human activity and resource use and the impact those activities and uses have on ecological systems" and "considers the ways in which individuals and communities respond to risks" | Adger et al. 2005, Nayak and Berkes 2014, Brown and Westaway 2011, Alexander 2013, Spector et al. 2019 |
| Dalgliesh et al., 2016 | No definition |  |
| Daniele et al., 2022 | Capacity of a system to keep delivering its essential functions despite challenges | Meuwissen et al. 2019 |
| Dardonville et al., 2022 | “Ability to ensure provision of system functions” | Meuwissen et al., 2019 |
| Daugstad, 2019 | "Farmer’s capability to navigate change in relations that are never stable" mobilizing buffer, adaptive and transformative capability | Wilson et al. 2018; Schermer et al. 2016; Darnhofer 2014 |
| Diserens et al., 2018 | "Resilience is the ability of a system to recover, reorganize and evolve following external stresses and disturbances" | Choptiany et al. 2015 |
| Dobkowtiz et al., 2020 | "opposite of vulnerability" | Douxchamp et al. 2017 |
| Duncan et al., 2017 | "capacity, possessed by a unit, *e.g.* household, community, or country, to maintain or improve standards of living while facing an uncertain risk landscape" | Barrett and Constas 2014 |
| El Ayadi et al., 2021 | Capacity of a system to maintain its long-term functions, i.e. its capacity to provide fodder and water to herds, as well as its multipurpose character | Nettier et al. 2017 |
| Griffiths and Evans, 2015 | Set of "flexible responses to system shocks (), that enables them to ‘bounce forward’" through a "reorganization of the current farm system, often within the constraints of culture and tradition, but one which also incorporates actions and measures intended to limit future vulnerability" | Comfort et al. 2001; Cutter et al. 2008; IPCC 2014; Sinclair et al. 2014, Milestad and Darnhofer 2003 |
| Gugissa et al., 2022 | "Ability of a production system to recover, reorganize, and evolve following external stresses and disturbances" | Choptiany et al., 2015 |
| Hammond et al., 2013 | "amount of change a system can undergo, while retaining similar function and structure" through its "ability to organize itself and to increase its capacity to adapt to changing conditions in the socioeconomic and natural environments" | The Resilience Alliance 2010 |
| Haydari et al., 2024 | Capabilities and potentials in the face of shocks, accidents and natural disasters, including recover and return to a previous state after a disaster | Martini and Platania 2019; Adger et  al. 2005; Badri et  al. 2014; Keck and Sakdapolrak 2013; Pasteur 2011 |
| Heckelman et al., 2018 | "function of social and ecological integrative processes and outcomes that enhance adaptive capacity, augment mitigation potential and reduce the vulnerability of a farming system" | Cabell and Oelofse 2012; Miller et al. 2010 |
| Herrera and Kopainsky, 2023 | " System’s ability to maintain its functionality even when it is affected by external disturbances" | Folke et al., 2010; C. Holling, 1986; Meuwissen et al., 2019 |
| Hossard et al., 2021 | "capacity to reorganize and maintain interconnected functions and structures that are defined at different temporal and spatial scales" | Walker et al. 2004; Souissi et al. 2018 |
| Jacobi et al., 2015 | "ability of a social–ecological system to withstand stress factors while maintaining its productivity, its main functions, and its capacities to learn and to adapt" | Adger 2003; Folke et al. 2010 |
| Jamil et al., 2021 | Capacity to increase farm income and enhance resource use efficiency |  |
| Jiri et al., 2017 | No definition |  |
| Khan et al., 2021 | "Capability to respond to the current or potential changes in the climate" - Synonym of adaptability |  |
| Kozicka et al., 2020 | recovery of the farm's performance as a results of the farmer's reconfiguration |  |
| Kuchimanchi et al., 2021 | "Changes a system can undergo due to exposure while continuing to remain within the set of naturalor desirable states [...] Resilience is the dynamic result of the response of an SES to a perturbation, which can be short-term coping or long-term adjustment adaptation" | Adger 2006 |
| Le Goff et al., 2022 | "the capacity of social, economic, and environmental systems to cope with a hazardous event or trend or disturbance, responding or reorganizing in ways that maintain their essential function, identity, and structure, while also maintaining the capacity for adaptation, learning, and transformation” | IPCC, 2014 |
| McKenzie et al., 2024 | No definition | No definition |
| McRae, 2024 | No definition | No definition |
| Melvani et al., 2020 | "Ability of a social or ecological system to absorb disturbances while retaining the same basic structure and ways of functioning, the capacity for selforganisation, and the capacity to adapt to stress and change and to endure" | Intergovernmental Panel on Climate Change 2007; Hoang et al. 2014 |
| Mohammed et al., 2021 | "socialecological outcome involving the interaction of complex ecological, socioeconomic, political, and cultural factors" | Folke 2006; Holling 1973 |
| Monjardino et al., 2022 | No definition |  |
| Mutabazi et al., 2015 | "ability of the farming system to withstand the shocks and stresses from climatic vagaries" |  |
| Mzyece et al., 2020 | Income stability |  |
| Nera et al., 2020 | "Capacity of a system to perform its functions, related to both private and public goods. A resilient system should be able to maintain the delivery of private and public goods in the face of shocks and persistent pressures" | Holling 1973; Urruty et al. 2016; Walker et al. 2004 |
| Nguyen et al., 2020 | No definition |  |
| Nyong et al., 2020 | "Desired pahtway of development" that allows farming system to face shocks and stresses | Gordon 2009 |
| Perin and Enahoro, 2023 | No definition | No definition |
| Poelma et al., 2021 | "the capacity of all people across generations to sustain and improve their livelihood opportunities and well-being despite environmental, economic, social and political disturbances" | Tanner et al. 2015, p.23 |
| Postigo et al., 2024 | Capacity of systems to "maintain their organization, structure, and function after a disturbance or transform when the situation is untenable to continue on the desired trajectories" | Folke, 2006; Walker et al., 2004, Chaigneau et al., 2022 |
| Poulton et al., 2016 | No definition |  |
| Rodriguez et al., 2011 | "ability of a farm business to absorb disturbances while remaining productive and profitable" | IPCC 2007 |
| Singh et al., 2020 | No definition |  |
| Son et al., 2021 | "capacity to deal with change and continue to develop" through "coping abilities to withstand, recover from stresses and surprises, and adapt to climate variability and change" |  |
| Souissi et al., 2018 | "ability to reorganize and maintain the function and structure of systems that are interconnected and cover different spatial and temporal scales" | Carpenter et al. 2001; Enfors and Gordon 2007; Walker et al. 2004 |
| Srinidhi et al., 2024 | "resilience is the capacity of a system to dynamically respond to, recover from and even thrive in changing climate conditions while continuing to maintain essential functions, identities and structures" | Dixon & Stringer, 2015; Rockefeller Foundation, 2009 |
| Taremwa et al., 2016 | No definition |  |
| Tran and Cook, 2024 | “capacity to live with complexity, uncertainty and change, and to continuously develop in the context of everchanging environments” | Folke et al., 2021 |
| Williams et al., 2020 | Capacity to maintain high levels of food security throughout and following a drought |  |
| Yessoufou et al., 2024 | “Resilience here covers the ability to cope with risk, to adapt, and to transform […], but also the ability to prepare before the risk occurs” | Folke et al. 2002 |

### Table S2: List of the 36 attributes used in the *attribute-based* and the *performance-based* body of literature.

|  | **Attribute category defined for the review** | **Attributes used by the authors in the studies of the systematic review** | **Attribute occurrence** | **References** |
| --- | --- | --- | --- | --- |
|  | Diversity | Number of crops and varieties | 40 | (Chapman et al. 2008; Rodriguez et al. 2011, 2014; Rufino et al. 2013; Lehmann et al. 2013; Lei et al. 2014; Mutabazi et al. 2015; Ghahramani and Moore 2016; Dalgliesh et al. 2016; Duncan et al. 2017; Mahmood et al. 2017; Paul et al. 2017; Descheemaeker et al. 2018; Souissi et al. 2018; Kabir et al. 2018; Ghahramani and Bowran 2018; Mutenje et al. 2019; Vigani and Kathage 2019; Kozicka et al. 2020; Omerkhil et al. 2020; Williams et al. 2020a; Cann et al. 2020; Mzyece and Ng’ombe 2020; Hossard et al. 2021; Mohammed et al. 2021; Monjardino et al. 2021, 2022; Carauta et al. 2021; Homann-Kee Tui et al. 2021; Amadu et al. 2021; Maggio et al. 2022; Rai et al. 2022; Dardonville et al. 2022; Asrari et al. 2022; Mugandani et al. 2022; Adhikari et al. 2023; Baccar et al. 2023; Rezgui et al. 2024; Alcon et al. 2024) |
|  |  | Number of crops, varieties, plants and species | 10 | (Martin and Magne 2015; Williams et al. 2018; Asmare et al. 2022; Bergez et al. 2022; Monjardino et al. 2022; Chaudhary et al. 2022; Soltani and Mellah 2023; Garba et al. 2023; Marie Chimi et al. 2023; Young et al. 2024) |
|  |  | Number of plants and species | 7 | (Millar et al. 2009; Jacobi et al. 2015; Duranton and Matthew 2018; Dobkowitz et al. 2020; Nyong et al. 2020; Melvani et al. 2020; Pinsard et al. 2023) |
|  |  | Number of livestock species and races | 4 | (Toni and Holanda Jr. 2008; Millar et al. 2009; Dobkowitz et al. 2020; Castaño-Sánchez et al. 2023) |
|  | Modularity | Volume of off-farm activity | 16 | (Komarek et al. 2012; Rodriguez et al. 2014; Mutabazi et al. 2015; Ghahramani and Moore 2016; Tibesigwa et al. 2017; Lopez-Ridaura et al. 2018; Souissi et al. 2018; Ghahramani and Bowran 2018; Ghahramani et al. 2020; Williams et al. 2020a; Khan et al. 2021; Bell et al. 2021; Rai et al. 2022; Chawdhery et al. 2022; Mugandani et al. 2022; Abdollahzadeh et al. 2023; Gemeda et al. 2023) |
|  |  | Number of cropping and livestock activities | 13 | (Jacobi et al. 2015; Mutabazi et al. 2015; Duncan et al. 2017; Rigolot et al. 2017; Lopez-Ridaura et al. 2018; Williams et al. 2020a, b; Melvani et al. 2020; Panta et al. 2020; Mishra et al. 2021; Khan et al. 2021; Mugandani et al. 2022; Monjardino et al. 2022) |
|  |  | Number of production activities | 9 | (Rotz et al. 2016; Vigani and Kathage 2019; Dobkowitz et al. 2020; Omerkhil et al. 2020; Ben Nasr et al. 2021; Hossard et al. 2021; Amadu et al. 2021; Koch et al. 2022; Asrari et al. 2022; Bhowmik et al. 2023) |
|  |  | Number of income generating activities | 5 | (Rufino et al. 2013; Rai et al. 2022; Abdollahzadeh et al. 2023; Marie Chimi et al. 2023; Postigo et al. 2024) |
|  |  | Forestry production | 1 | (Djanibekov and Khamzina 2016) |
|  | Openness | Access to irrigation | 31 | (Schaap et al. 2013; Rodriguez et al. 2014; Mutabazi et al. 2015; Poulton et al. 2016; Hochman et al. 2017; Tibesigwa et al. 2017; Lopez-Ridaura et al. 2018; Souissi et al. 2018; Dobkowitz et al. 2020; Omerkhil et al. 2020; Singh et al. 2020; Williams et al. 2020a; Ben Nasr et al. 2021; Hossard et al. 2021; Jamil et al. 2021; Mishra et al. 2021; Khan et al. 2021, 2022; Dardonville et al. 2022; Varadan et al. 2022; Asmare et al. 2022; Bergez et al. 2022; Chawdhery et al. 2022; Abdollahzadeh et al. 2023; Soltani and Mellah 2023; Adhikari et al. 2023; Gemeda et al. 2023; Collins et al. 2023; Marie Chimi et al. 2023; Kumar et al. 2024; Alotaibi et al. 2024) |
|  |  | Access to information and advice | 26 | (Jacobi et al. 2015; Mutabazi et al. 2015; Jiri et al. 2017; Tibesigwa et al. 2017; Dobkowitz et al. 2020; Omerkhil et al. 2020; Williams et al. 2020a, b; Panta et al. 2020; Ben Nasr et al. 2021; Mohammed et al. 2021; Naz and Saqib 2021; Khan et al. 2021; Chukwuone and Amaechina 2021; Rai et al. 2022; Gugissa et al. 2022; Koch et al. 2022; Asrari et al. 2022; Mugandani et al. 2022; Abdollahzadeh et al. 2023; Gemeda et al. 2023; Haydari et al. 2023; Marie Chimi et al. 2023; Amarnath et al. 2023; Postigo et al. 2024; Alotaibi et al. 2024) |
|  |  | Access to credit and insurance | 21 | (Mutabazi et al. 2015; Jiri et al. 2017; Tibesigwa et al. 2017; Kath et al. 2018; Vigani and Kathage 2019; Dobkowitz et al. 2020; Omerkhil et al. 2020; Williams et al. 2020a; Panta et al. 2020; Hossard et al. 2021; Mohammed et al. 2021; Khan et al. 2021; Rai et al. 2022; Asrari et al. 2022; Abdollahzadeh et al. 2023; Soltani and Mellah 2023; Gemeda et al. 2023; Marie Chimi et al. 2023; Amarnath et al. 2023; Postigo et al. 2024; Alotaibi et al. 2024) |
|  |  | Amount of mineral fertilizer | 19 | (Keil et al. 2009; Bobojonov and Aw-Hassan 2014; Mwinuka et al. 2016; Ghahramani and Moore 2016; Poulton et al. 2016; Rigolot et al. 2017; Descheemaeker et al. 2018; Kabir et al. 2018; Kadigi et al. 2020; Monjardino et al. 2021; Homann-Kee Tui et al. 2021; Maggio et al. 2022; Dardonville et al. 2022; Varadan et al. 2022; Chaudhary et al. 2022; Abdollahzadeh et al. 2023; Pinsard et al. 2023; Kumar et al. 2024) |
|  |  | Access to improved varieties | 16 | (Keil et al. 2009; Mutabazi et al. 2015; Dalgliesh et al. 2016; Poulton et al. 2016; Ghahramani and Bowran 2018; Omerkhil et al. 2020; Kadigi et al. 2020; Jamil et al. 2021; Monjardino et al. 2021; Homann-Kee Tui et al. 2021; Asmare et al. 2022; Mugandani et al. 2022; Chaudhary et al. 2022; Abdollahzadeh et al. 2023; Amarnath et al. 2023; Kumar et al. 2024) |
|  |  | Access to selling market | 7 | (Dobkowitz et al. 2020; Hossard et al. 2021; Naz and Saqib 2021; Maggio et al. 2022; Gugissa et al. 2022; Herrera and Kopainsky 2023; Kumar et al. 2024) |
|  |  | Volume of subsidies | 7 | (Lehmann et al. 2013; Mahmood et al. 2017; Dobkowitz et al. 2020; Omerkhil et al. 2020; Shrestha et al. 2020; Williams et al. 2020a; Hossard et al. 2021) |
|  |  | Access to purchased feed | 6 | (Ho et al. 2015; Rigolot et al. 2017; Bilotto et al. 2019, 2021; Monjardino et al. 2021; Pinsard et al. 2023) |
|  |  | Access to agricultural and grazing lands | 6 | (Gugissa et al. 2022; Mugandani et al. 2022; Soltani and Mellah 2023; Bhowmik et al. 2023; Herrera and Kopainsky 2023; Gemeda et al. 2023) |
|  |  | Access to public and private development | 6 | (Homann-Kee Tui et al. 2021; Rai et al. 2022; Asrari et al. 2022; Mugandani et al. 2022; Abdollahzadeh et al. 2023; Kumar et al. 2024) |
|  |  | Amount of mineral herbicide / pesticide | 6 | (Mutabazi et al. 2015; Ghahramani and Bowran 2018; Omerkhil et al. 2020; Monjardino et al. 2021) |
|  |  | Access to water of quality | 4 | (Mugandani et al. 2022; Gemeda et al. 2023; Postigo et al. 2024; Alotaibi et al. 2024) |
|  |  | Access to production contract | 1 | (Vigani and Kathage 2019) |
|  |  | Access to feed and fuel from natural habitat | 1 | (Kumar et al. 2024) |
|  | Reserves | Land size | 23 | (Keil et al. 2009; Mutabazi et al. 2015; Ghahramani and Moore 2016; Duncan et al. 2017; Jiri et al. 2017; Lopez-Ridaura et al. 2018; Souissi et al. 2018; Omerkhil et al. 2020; Shrestha et al. 2020; Panta et al. 2020; Ben Nasr et al. 2021; Hossard et al. 2021; Mohammed et al. 2021; Naz and Saqib 2021; Khan et al. 2021; Maggio et al. 2022; Varadan et al. 2022; Chawdhery et al. 2022; Abdollahzadeh et al. 2023; Gemeda et al. 2023; Marie Chimi et al. 2023; Postigo et al. 2024; Alotaibi et al. 2024) |
|  |  | Labour stock | 18 | (Keil et al. 2009; Rodriguez et al. 2011; Mutabazi et al. 2015; Jiri et al. 2017; Lopez-Ridaura et al. 2018; Souissi et al. 2018; Omerkhil et al. 2020; Ben Nasr et al. 2021; Hossard et al. 2021; Mishra et al. 2021; Mohammed et al. 2021; Khan et al. 2021; Chukwuone and Amaechina 2021; Abdollahzadeh et al. 2023; Soltani and Mellah 2023; Kumar et al. 2024; Postigo et al. 2024; Alotaibi et al. 2024) |
|  |  | Stocking density | 16 | (Millar et al. 2009; Komarek et al. 2012; Scott 2013; Phelan et al. 2015; Iglesias et al. 2016; Ghahramani and Moore 2016; Lopez-Ridaura et al. 2018; Descheemaeker et al. 2018; Bilotto et al. 2019, 2021; Shrestha et al. 2020; Homann-Kee Tui et al. 2021; Varadan et al. 2022; Monjardino et al. 2022; Pinsard et al. 2023) |
|  |  | Soil fertility | 16 | (Schaap et al. 2013; Tibesigwa et al. 2017; Descheemaeker et al. 2018; Souissi et al. 2018; Dobkowitz et al. 2020; Mishra et al. 2021; Homann-Kee Tui et al. 2021; Gugissa et al. 2022; Dardonville et al. 2022; Koch et al. 2022; Abdollahzadeh et al. 2023; Bhowmik et al. 2023; Marie Chimi et al. 2023; Kumar et al. 2024; Postigo et al. 2024; Alotaibi et al. 2024) |
|  |  | Number of livestock heads | 15 | (Duncan et al. 2017; Jiri et al. 2017; Amamou et al. 2018; Lopez-Ridaura et al. 2018; Descheemaeker et al. 2018; Souissi et al. 2018; Dobkowitz et al. 2020; Shrestha et al. 2020; Hossard et al. 2021; Monjardino et al. 2021; Naz and Saqib 2021; Soltani and Mellah 2023; Marie Chimi et al. 2023; Kumar et al. 2024; Alotaibi et al. 2024) |
|  |  | Amount of liquidity reserves | 10 | (Phelan et al. 2015; Jiri et al. 2017; Dobkowitz et al. 2020; Hossard et al. 2021; Naz and Saqib 2021; Asrari et al. 2022; Abdollahzadeh et al. 2023; Marie Chimi et al. 2023; Kumar et al. 2024; Postigo et al. 2024) |
|  |  | Degree of resources endowment | 9 | (Keil et al. 2009; Rigolot et al. 2017; Descheemaeker et al. 2018; Kabir et al. 2020; Homann-Kee Tui et al. 2021; Maggio et al. 2022; Koch et al. 2022; Bhowmik et al. 2023; Kumar et al. 2024) |
|  |  |  |  |  |
|  |  | Number of mechanized equipment | 5 | (Lopez-Ridaura et al. 2018; Ben Nasr et al. 2021; Abdollahzadeh et al. 2023; Gemeda et al. 2023; Marie Chimi et al. 2023) |
|  |  | Groundwater level | 1 | (Bergez et al. 2022) |
|  | Tightness of feedbacks | Feed produced for livestock | 1 | (Lurette et al. 2013; Martin and Magne 2015; Phelan et al. 2015; Iglesias et al. 2016; Lopez-Ridaura et al. 2018; Ghahramani and Bowran 2018; Ghahramani et al. 2020; Varadan et al. 2022; Monjardino et al. 2022; Pinsard et al. 2023; Sanou et al. 2023) |
|  |  | Crop residues used as fodder | 4 | (Rigolot et al. 2017; Monjardino et al. 2021; Homann-Kee Tui et al. 2021; Sanou et al. 2023) |
|  |  | Manure used as organic fertilizer | 3 | (Descheemaeker et al. 2018; Homann-Kee Tui et al. 2021; Maggio et al. 2022) |
|  |  | Crop residues used as mulch | 2 | (Rigolot et al. 2017; Monjardino et al. 2021) |
|  |  | Crop residues uses | 2 | (Lopez-Ridaura et al. 2018; Asmare et al. 2022) |
|  |  | Number of types of use of manure | 1 | (Rotz et al. 2016) |

### Table S3: List of the 204 studies systematically reviewed from Scopus and Web of Science databases on 13/08/2024

| **Authors** | **Title** | **Year** |
| --- | --- | --- |
| Abdollahzadeh G.; Sharifzadeh M.S.; Sklenička P.; Azadi H. | Adaptive capacity of farming systems to climate change in Iran: Application of composite index approach | 2023 |
| Adelhart Toorop R.; Lopez-Ridaura S.; Jat M.L.; Eichenseer P.; Bijarniya D.; Jat R.K.; Groot J.C.J. | Analyzing antifragility among smallholder farmers in Bihar, India: An assessment of farmers' vulnerability and the strengths of positive deviants | 2023 |
| Adhikari L.; Komarek A.M.; de Voil P.; Rodriguez D. | A framework for the assessment of farm diversification options in broadacre agriculture | 2023 |
| Ado, AM; Leshan, J; Savadogo, P; Bo, L; Shah, AA | Farmers' awareness and perception of climate change impacts: case study of Aguie district in Niger | 2019 |
| Ado, AM; Savadogo, P; Pervez, AKMK; Mudimu, GT | Farmers' perceptions and adaptation strategies to climate risks and their determinants: insights from a farming community of Aguie district in Niger | 2020 |
| Ahmed N., Diana J.S., | Threatening "white gold": Impacts of climate change on shrimp farming in coastal Bangladesh | 2015 |
| Ahmed Z., Guha G.S., Shew A.M., Alam G.M.M., | Climate change risk perceptions and agricultural adaptation strategies in vulnerable riverine char islands of Bangladesh | 2021 |
| Albert M.; Bergez J.-E.; Willaume M.; Couture S. | Vulnerability of Maize Farming Systems to Climate Change: Farmers’ Opinions Differ about the Relevance of Adaptation Strategies | 2022 |
| Alcon F.; Albaladejo-García J.A.; Martínez-García V.; Rossi E.S.; Blasi E.; Lehtonen H.; Martínez-Paz J.M.; Zabala J.A. | Cost benefit analysis of diversified farming systems across Europe: Incorporating non-market benefits of ecosystem services | 2024 |
| Alotaibi B.A.; Xu W.; Shah A.A.; Ullah W. | Exploring Climate-Induced Agricultural Risk in Saudi Arabia: Evidence from Farming Communities of Medina Region | 2024 |
| Amadu F.O.; McNamara P.E.; Davis K.E. | Soil health and grain yield impacts of climate resilient agriculture projects: Evidence from southern Malawi | 2021 |
| Amamou H.; Mahouachi M.; Dale L.M.; Beckers Y.; Hammami H. | Vulnerability assessments in dairy cattle farms based on individual sensitivity to heat stress | 2022 |
| Amamou, H; Ben Sassi, M; Aouadi, H; Khemiri, H; Mahouachi, M; Beckers, Y; Hammami, H | Climate change-related risks and adaptation strategies as perceived in dairy cattle farming systems in Tunisia | 2018 |
| Amarnath G.; Taron A.; Alahacoon N.; Ghosh S. | Bundled climate-smart agricultural solutions for smallholder farmers in Sri Lanka | 2023 |
| Antwi K.; Antwi-Agyei P. | Intra-gendered perceptions and adoption of climate-smart agriculture: Evidence from smallholder farmers in the Upper East Region of Ghana | 2023 |
| Aryal J.P., Sapkota T.B., Rahut D.B., Krupnik T.J., Shahrin S., Jat M.L., Stirling C.M., | Major Climate risks and Adaptation Strategies of Smallholder Farmers in Coastal Bangladesh | 2020 |
| Asmare F.; Jaraitė J.; Kažukauskas A. | Climate change adaptation and productive efficiency of subsistence farming: A bias-corrected panel data stochastic frontier approach | 2022 |
| Asrari A.; Omidi Najafabadi M.; Farajollah Hosseini J. | Modeling resilience behavior against climate change with food security approach | 2022 |
| Awazi N.P.; Temgoua L.F.; Tientcheu-Avana M.-L.; Tchamba M.N. | Reducing Vulnerability to Climate Change Through Agroforestry: Case Study of Small-Scale Farmers in the Northwest Region of Cameroon | 2023 |
| Baccar, M; Raynal, H; Sekhar, M; Bergez, JE; Willaume, M; Casel, P; Giriraj, P; Murthy, S; Ruiz, L | Dynamics of crop category choices reveal strategies and tactics used by smallholder farmers in India to cope with unreliable water availability | 2023 |
| Bagambilana F.R.; Rugumamu W.M. | Small-Scale Farmers’ Vulnerability to Biophysical and Socio-Economic Risks in Semi-Arid Lowlands of Mwanga District, Kilimanjaro Region, Tanzania | 2023 |
| Bagambilana F.R.; Rugumamu W.M. | Determinants of Farmers’ Adaptation Intent And Adoption of Adaptation Strategies To Climate Change And Variability In Mwanga District, Tanzania | 2023 |
| Bardati D., | Participatory Agroecological Assessment of Farmers’ Capacity to Adapt to Climate Change in Malawi | 2019 |
| Bardsley D.K., Palazzo E., Pütz M., | Regional path dependence and climate change adaptation: A case study from the McLaren Vale, South Australia | 2018 |
| Bell L.W., Moore A.D., Thomas D.T., | Diversified crop-livestock farms are risk-efficient in the face of price and production variability | 2021 |
| Below T.B., Schmid J.C., Sieber S., | Farmers’ knowledge and perception of climatic risks and options for climate change adaptation: a case study from two Tanzanian villages | 2015 |
| Ben Nasr J., Chaar H., Bouchiba F., Zaibet L., | Assessing and building climate change resilience of farming systems in Tunisian semi-arid areas | 2021 |
| Bergez, JE; Baccar, M; Sekhar, M; Ruiz, L | NIRAVARI: A Parsimonious Bio-Decisional Model for Assessing the Sustainability and Vulnerability of Rainfed or Groundwater-Irrigated Farming Systems in Indian Agriculture | 2022 |
| Bhowmik B.C.; Rima N.N.; Gosh K.; Hossain M.A.; Murray F.J.; Little D.C.; Mamun A.-A. | Salinity extrusion and resilience of coastal aquaculture to the climatic changes in the southwest region of Bangladesh | 2023 |
| Bhuyan M.I.; Supit I.; Kumar U.; Mia S.; Ludwig F. | The significance of farmers’ climate change and salinity perceptions for on-farm adaptation strategies in the south-central coast of Bangladesh | 2024 |
| Bilotto F., Recavarren P., Vibart R., Machado C.F., | Backgrounding strategy effects on farm productivity, profitability and greenhouse gas emissions of cow-calf systems in the Flooding Pampas of Argentina | 2019 |
| Bilotto F., Vibart R., Wall A., Machado C.F., | Estimation of the inter-annual marginal value of additional feed and its replacement cost for beef cattle systems in the Flooding Pampas of Argentina | 2021 |
| Bobojonov I., Aw-Hassan A., | Impacts of climate change on farm income security in Central Asia: An integrated modeling approach | 2014 |
| Cann, DJ; Hunt, JR; Malcolm, B | Long fallows can maintain whole-farm profit and reduce risk in semi-arid south-eastern Australia | 2020 |
| Carauta M., Parussis J., Hampf A., Libera A., Berger T., | No more double cropping in Mato Grosso, Brazil? Evaluating the potential impact of climate change on the profitability of farm systems | 2021 |
| Castaño-Sánchez, JP; Rotz, CA; McIntosh, MM; Tolle, C; Gifford, CA; Duff, GC; Spiegal, SA | Grass finishing of Criollo cattle can provide an environmentally preferred and cost effective meat supply chain from United States drylands | 2023 |
| Chapman D.F., Kenny S.N., Beca D., Johnson I.R., | Pasture and forage crop systems for non-irrigated dairy farms in southern Australia. 2. Inter-annual variation in forage supply, and business risk | 2008 |
| Chaudhary B.R.; Erskine W.; Acciaioli G. | Hybrid knowledge and climate-resilient agriculture practices of the Tharu in the western Tarai, Nepal | 2022 |
| Chawdhery, MRA; Al-Mueed, M; Wazed, MA; Emran, SA; Chowdhury, MAH; Hussain, SG | Climate Change Impacts Assessment Using Crop Simulation Model Intercomparison Approach in Northern Indo-Gangetic Basin of Bangladesh | 2022 |
| Chukwuone N.A.; Amaechina E.C. | Factors affecting climate change coping strategies used by smallholder farmers under root crop farming systems in derived savannah ecology zone of Nigeria | 2021 |
| Ciftcioglu, GC | Assessment of the resilience of the agricultural landscapes and associated ecosystem services at multiple scales (a farm and landscape) in Kyrenia (Girne) Region of Northern Cyprus | 2022 |
| Collins B.; Attard S.; Banhalmi-Zakar Z.; Everingham Y. | i-RAT: A discussion support system to rapidly assess economic and environmental impacts resulting from different sugarcane irrigation practices | 2023 |
| Córdova R., Hogarth N.J., Kanninen M., | Mountain farming systems' exposure and sensitivity to climate change and variability: Agroforestry and conventional agriculture systems compared in Ecuador's Indigenous Territory of Kayambi people | 2019 |
| Cradock-Henry N.A., | Linking the social, economic, and agroecological: A resilience framework for dairy farming | 2021 |
| Dahal K.R.; Dahal P.; Adhikari R.K.; Naukkarinen V.; Panday D.; Bista N.; Helenius J.; Marambe B. | Climate Change Impacts and Adaptation in a Hill Farming System of the Himalayan Region: Climatic Trends, Farmers’ Perceptions and Practices | 2023 |
| Dalgliesh, NP; Charlesworth, P; Lonh, L; Poulton, PL | Promoting resilience in Cambodian lowland rice ecosystems-Farming system research to support flexible climate response strategies for smallholder farmers | 2016 |
| Dam T.H.T., Tur-Cardona J., Speelman S., Amjath-Babu T.S., Sam A.S., Zander P., | Incremental and transformative adaptation preferences of rice farmers against increasing soil salinity - Evidence from choice experiments in north central Vietnam | 2021 |
| Daniele B.-C.; Barbara S.; Isabel B.; Alberto G. | Analysis of perceived robustness, adaptability and transformability of Spanish extensive livestock farms under alternative challenging scenarios | 2022 |
| Dardonville M.; Bockstaller C.; Villerd J.; Therond O. | Resilience of agricultural systems: biodiversity-based systems are stable, while intensified ones are resistant and high-yielding | 2022 |
| Datta P.; Behera B. | What caused smallholders to change farming practices in the era of climate change? Empirical evidence from Sub-Himalayan West Bengal, India | 2022 |
| Daugstad, K | Resilience in Mountain Farming in Norway | 2019 |
| Descheemaeker K., Zijlstra M., Masikati P., Crespo O., Homann-Kee Tui S., | Effects of climate change and adaptation on the livestock component of mixed farming systems: A modelling study from semi-arid Zimbabwe | 2018 |
| Diserens, F; Choptiany, JMH; Barjolle, D; Graeub, B; Durand, C; Six, J | Resilience Assessment of Swiss Farming Systems: Piloting the SHARP-Tool in Vaud | 2018 |
| Djanibekov, U; Khamzina, A | Stochastic Economic Assessment of Afforestation on Marginal Land in Irrigated Farming System | 2016 |
| Dobkowitz S., Walz A., Baroni G., Pérez-Marin A.M., | Cross-scale vulnerability assessment for smallholder farming: A case study from the northeast of Brazil | 2020 |
| Duncan J.M., Tompkins E.L., Dash J., Tripathy B., | Resilience to hazards: Rice farmers in the Mahanadi Delta, India | 2017 |
| Duranton, C; Matthew, C | Impact of introducing a herb pasture area into a New Zealand sheep and beef hill country farm system: a modeling analysis | 2018 |
| Eakin H., Tucker C.M., Castellanos E., Diaz-Porras R., Barrera J.F., Morales H., | Adaptation in a multi-stressor environment: Perceptions and responses to climatic and economic risks by coffee growers in Mesoamerica | 2014 |
| El Aayadi S.; Araba A.; Jouven M. | Resilience of the pastoral component of Moroccan small ruminant systems in mountain areas | 2021 |
| Engler A.; Rotman M.L.; Poortvliet P.M. | Farmers’ perceived vulnerability and proactive versus reactive climate change adaptation in chile’s maule region | 2021 |
| Esham M., Garforth C., | Agricultural adaptation to climate change: Insights from a farming community in Sri Lanka | 2013 |
| Faisal M., Chunping X., Abbas A., Raza M.H., Akhtar S., Ajmal M.A., Ali A., | Do risk perceptions and constraints influence the adoption of climate change practices among small livestock herders in Punjab, Pakistan? | 2021 |
| Furoc-Paelmo, R; Cosico, RSA; Cabahug, RED; Castillo, AKA; Castillo, ASA; Visco, RG | Farmers' Perception on the Sustainability of as a Climate Change Adaptation Strategy in Agusan Agusan Del Sur and North Cotobato, Philippines | 2018 |
| Garba I.I.; Bell L.W.; Chapman S.C.; deVoil P.; Kamara A.Y.; Williams A. | Modelling the impacts of diverse cover crops on soil water and nitrogen and cash crop yields in a sub-tropical dryland | 2023 |
| Gemeda D.O.; Korecha D.; Garedew W. | Climate Change Perception and Vulnerability Assessment of the Farming Communities in the Southwest Parts of Ethiopia | 2023 |
| Ghahramani A., Bowran D., | Transformative and systemic climate change adaptations in mixed crop-livestock farming systems | 2018 |
| Ghahramani A., Kingwell R.S., Maraseni T.N., | Land use change in Australian mixed crop-livestock systems as a transformative climate change adaptation | 2020 |
| Ghahramani A., Moore A.D., | Impact of climate changes on existing crop-livestock farming systems | 2016 |
| Griffiths R., Evans N., | The welsh marches: Resilient farmers? Exploring farmers' resilience to extreme weather events in the recent past | 2015 |
| Gugissa D.A.; Abro Z.; Tefera T. | Achieving a Climate-Change Resilient Farming System through Push–Pull Technology: Evidence from Maize Farming Systems in Ethiopia | 2022 |
| Hammond B., Berardi G., Green R., | Resilience in agriculture: Smalland medium-sized farms in northwest Washington State | 2013 |
| Haydari H.; Agahi H.; Geravandi S. | Social Resilience of Iranian Potato Farmers Against Climate Changes | 2024 |
| Hebbsale Mallappa V.K., Shivamurthy M., | Factor influencing fishery-based farmers’ perception and their response to climate-induced crisis management. | 2021 |
| Heckelman, A; Smukler, S; Wittman, H | Cultivating climate resilience: a participatory assessment of organic and conventional rice systems in the Philippines | 2018 |
| Herrera H.; Kopainsky B. | Using microworlds for policymaking in the context of resilient farming systems | 2023 |
| Ho C.K.M., Malcolm B., Doyle P.T., | Supplementary feeding options to alleviate the impacts of decreased water availability on dairy-farm economic performance in northern Victoria | 2015 |
| Hochman Z., Horan H., Reddy D.R., Sreenivas G., Tallapragada C., Adusumilli R., Gaydon D., Singh K.K., Roth C.H., | Smallholder farmers managing climate risk in India: 1. Adapting to a variable climate | 2017 |
| Hossard L., Fadlaoui A., Ricote E., Belhouchette H., | Assessing the resilience of farming systems on the Saïs plain, Morocco | 2021 |
| Hussain W.; Khan M.A. | Climate change-induced Glacial Lake Outburst Floods in Hunza Valley of Pakistan: an assessment of indigenous farming community perceptions and adaptation | 2024 |
| Iglesias E., Báez K., Diaz-Ambrona C.H., | Assessing drought risk in Mediterranean Dehesa grazing lands | 2016 |
| Jacobi J., Schneider M., Pillco Mariscal M., Huber S., Weidmann S., Bottazzi P., Rist S., | Farm Resilience in Organic and Nonorganic Cocoa Farming Systems in Alto Beni, Bolivia | 2015 |
| Jamal, MR; Kristiansen, P; Kabir, MJ; de Bruyn, LL | Risks and adaptation dynamics in shrimp and prawn-based farming systems in southwest coastal Bangladesh | 2023 |
| Jamil I., Jun W., Mughal B., Raza M.H., Imran M.A., Waheed A., | Does the adaptation of climate-smart agricultural practices increase farmers’ resilience to climate change? | 2021 |
| Jezeer R.E., Verweij P.A., Boot R.G.A., Junginger M., Santos M.J., | Influence of livelihood assets, experienced shocks and perceived risks on smallholder coffee farming practices in Peru | 2019 |
| Jiri O., Mafongoya P.L., Chivenge P., | Building climate change resilience through adaptation in smallholder farming systems in semi-arid Zimbabwe | 2017 |
| Joshi, B; Ji, WH; Joshi, NB | Farm households' perception on climate change and adaptation practices A case from mountain district of Nepal | 2017 |
| Kabir M.J., Cramb R., Gaydon D.S., Roth C.H., | Bio-economic evaluation of cropping systems for saline coastal Bangladesh: III Benefits of adaptation in current and future environments | 2018 |
| Kabir, J; Cramb, R; Alauddin, M; Gaydon, DS; Roth, CH | Farmers' perceptions and management of risk in rice/shrimp farming systems in South-West Coastal Bangladesh | 2020 |
| Kadigi I.L., Mutabazi K.D., Philip D., Richardson J.W., Bizimana J.-C., Mbungu W., Mahoo H.F., Sieber S., | An economic comparison between alternative rice farming systems in Tanzania using a monte carlo simulation approach | 2020 |
| Kalele D.N.; Ogara W.O.; Oludhe C.; Onono J.O. | Climate change impacts and relevance of smallholder farmers’ response in arid and semi-arid lands in Kenya | 2021 |
| Kath J., Mushtaq S., Henry R., Adeyinka A., Stone R., | Index insurance benefits agricultural producers exposed to excessive rainfall risk | 2018 |
| Keil A., Teufel N., Gunawan D., Leemhuis C., | Vulnerability of smallholder farmers to ENSO-related drought in Indonesia | 2009 |
| Khan N.A.; Gong Z.; Shah A.A. | Synergy between climate risk perception, adaptation responses, and agricultural productivity: the case of rice farming communities in Pakistan | 2022 |
| Khan N.A.; Shah A.A.; Chowdhury A.; Tariq M.A.U.R.; Khanal U. | Rice farmers’ perceptions about temperature and rainfall variations, respective adaptation measures, and determinants: Implications for sustainable farming systems | 2022 |
| Khan, NA; Gao, QJ; Abid, M; Shah, AA | Mapping farmers' vulnerability to climate change and its induced hazards: evidence from the rice-growing zones of Punjab, Pakistan | 2021 |
| Kmoch L., Pagella T., Palm M., Sinclair F., | Using local agroecological knowledge in climate change adaptation: A study of tree-based options in Northern Morocco | 2018 |
| Koch O.; Ibisch P.L.; Bloch R. | Climate change and shifting land-use: Consequences for smallholder agroforestry systems and rural livelihoods in the southwest Ethiopian Highlands | 2022 |
| Komarek A.M., McDonald C.K., Bell L.W., Whish J.P.M., Robertson M.J., MacLeod N.D., Bellotti W.D., | Whole-farm effects of livestock intensification in smallholder systems in Gansu, China | 2012 |
| Kozicka M., Gotor E., Ocimati W., de Jager T., Kikulwe E., Groot J.C.J., | Responding to future regime shifts with agrobiodiversity: A multi-level perspective on small-scale farming in Uganda | 2020 |
| Kpenekuu F.; Antwi-Agyei P.; Nimoh F.; Osei M.A.; Guodaar L. | Understanding drought risk management in vulnerable communities in dryland farming systems: Evidence from northwest Ghana | 2024 |
| Kuchimanchi, BR; van Paassen, A; Oosting, SJ | Understanding the vulnerability, farming strategies and development pathways of smallholder farming systems in Telangana, India | 2021 |
| Kumar P.; Fürst C.; Joshi P.K. | Differentiated socio-ecological system approach for vulnerability and adaptation assessment in the Central Himalaya | 2024 |
| Le Goff U.; Sander A.; Lagana M.H.; Barjolle D.; Phillips S.; Six J. | Raising up to the climate challenge - Understanding and assessing farmers’ strategies to build their resilience. A comparative analysis between Ugandan and Swiss farmers | 2022 |
| Lehmann N., Briner S., Finger R., | The impact of climate and price risks on agricultural land use and crop management decisions | 2013 |
| Lei Y., Wang J., Yue Y., Yin Y., Sheng Z., | How adjustments in land use patterns contribute to drought risk adaptation in a changing climate-A case study in China | 2014 |
| Lopez-Ridaura S., Frelat R., van Wijk M.T., Valbuena D., Krupnik T.J., Jat M.L., | Climate smart agriculture, farm household typologies and food security: An ex-ante assessment from Eastern India | 2018 |
| Lurette, A; Aubron, C; Moulin, CH | A simple model to assess the sensitivity of grassland dairy systems to scenarios of seasonal biomass production variability | 2013 |
| Maggio G.; Mastrorillo M.; Sitko N.J. | Adapting to High Temperatures: Effect of Farm Practices and Their Adoption Duration on Total Value of Crop Production in Uganda | 2022 |
| Mahmood F., Belhouchette H., Nasim W., Shahzad T., Hussain S., Therond O., Fahad S., Refat Sultana S., Wery J., | Economic and environmental impacts of introducing grain legumes in farming systems of Midi-Pyrenees region (France): A simulation approach | 2017 |
| Mangaza L., Sonwa D.J., Batsi G., Ebuy J., Kahindo J.-M., | Building a framework towards climate-smart agriculture in the Yangambi landscape, Democratic Republic of Congo (DRC) | 2021 |
| Marie Chimi P.; Armand Mala W.; Ngamsou Abdel K.; Louis Fobane J.; Manga Essouma F.; Hermann Matick J.; Yldephonse Nyonce Pokam E.; Tcheferi I.; Martin Bell J. | Vulnerability of family farming systems to climate change: The case of the forest-savannah transition zone, Centre Region of Cameroon | 2023 |
| Martin G., Magne M.A., | Agricultural diversity to increase adaptive capacity and reduce vulnerability of livestock systems against weather variability - A farm-scale simulation study | 2015 |
| Mavhura E.; Manyangadze T.; Aryal K.R. | Perceived impacts of climate variability and change: an exploration of farmers’ adaptation strategies in Zimbabwe’s intensive farming region | 2022 |
| McKenzie D.K.; Joyce J.; Zander K.K.; Wurm P.A.S.; Caudwell K.M. | Eastern Australian Farmers Managing and Thinking Differently: Innovative Adaptation Cycles | 2024 |
| McRae S.M. | Agroforestry in temperate-climate commercial agriculture: Feedback from agroforestry practitioners in the Mid-Atlantic United States | 2024 |
| Melvani K., Bristow M., Moles J., Crase B., Kaestli M., | Multiple livelihood strategies and high floristic diversity increase the adaptive capacity and resilience of Sri Lankan farming enterprises | 2020 |
| Millar G.D., Jones R.E., Michalk D.L., Brady S., | An exploratory tool for analysis of forage and livestock production options | 2009 |
| Mishra A., Ketelaar J.W., Uphoff N., Whitten M., | Food security and climate-smart agriculture in the lower Mekong basin of Southeast Asia: evaluating impacts of system of rice intensification with special reference to rainfed agriculture | 2021 |
| Mohammed K., Batung E., Kansanga M., Nyantakyi-Frimpong H., Luginaah I., | Livelihood diversification strategies and resilience to climate change in semi-arid northern Ghana | 2021 |
| Mokoena O.P.; Ntuli T.S.; Ramarumo T.; Seeletse S.M. | Sustainability of Rural Small-Scale Farmers Using a Thematic Content-Fed Analytic Hierarchy Process | 2023 |
| Monjardino M., Ridaura S.L., Van Loon J., Mottaleb K., Kruseman G., Zepeda A., Hernández E.O., Burgueño J., Singh R.G., Govaerts B., Erenstein O., | Disaggregating the value of conservation agriculture to inform smallholder transition to sustainable farming: A mexican case study | 2021 |
| Monjardino M.; Loi A.; Thomas D.T.; Revell C.K.; Flohr B.M.; Llewellyn R.S.; Norman H.C. | Improved legume pastures increase economic value, resilience and sustainability of crop-livestock systems | 2022 |
| Morel K.; Cartau K. | Adaptation of organic vegetable farmers to climate change: An exploratory study in the Paris region | 2023 |
| Morton L.W., Roesch-McNally G., Wilke A.K., | Upper midwest farmer perceptions: Too much uncertainty about impacts of climate change to justify changing current agricultural practices | 2017 |
| Mubiru D.N., Radeny M., Kyazze F.B., Zziwa A., Lwasa J., Kinyangi J., Mungai C., | Climate trends, risks and coping strategies in smallholder farming systems in Uganda | 2018 |
| Mugandani R.; Muziri T.; Murewi C.T.F.; Mugadza A.; Chitata T.; Sungirai M.; Zirebwa F.S.; Manhondo P.; Mupfiga E.T.; Nyamutowa C.; Mudereri B.T.; Mugari Z.E.; Mwadzingeni L.; Mafongoya P. | Mapping and Managing Livelihoods Vulnerability to Drought: A Case Study of Chivi District in Zimbabwe | 2022 |
| Mulinde C., Majaliwa J.G.M., Twinomuhangi R., Mfitumukiza D., Komutunga E., Ampaire E., Asiimwe J., Van Asten P., Jassogne L., | Perceived climate risks and adaptation drivers in diverse coffee landscapes of Uganda | 2019 |
| Murungweni C., van Wijk M.T., Giller K.E., Andersson J.A., Smaling E.M.A., | Adaptive livelihood strategies employed by farmers to close the food gap in semi-arid south eastern Zimbabwe | 2014 |
| Mutabazi K.D., Amjath-Babu T.S., Sieber S., | Influence of livelihood resources on adaptive strategies to enhance climatic resilience of farm households in Morogoro, Tanzania: an indicator-based analysis | 2015 |
| Mutenje M.J., Farnworth C.R., Stirling C., Thierfelder C., Mupangwa W., Nyagumbo I., | A cost-benefit analysis of climate-smart agriculture options in Southern Africa: Balancing gender and technology | 2019 |
| Mwadzingeni L.; Mugandani R.; Mafongoya P.L. | Perception of climate change and coping strategies among smallholder irrigators in Zimbabwe | 2023 |
| Mwinuka, L; Mutabazi, KD; Makindara, J; Sieber, S | Reckoning the risks and rewards of fertilizer micro-dosing in a sub-humid farming system in Tanzania | 2016 |
| Mzyece, A; Ng'ombe, JN | Does Crop Diversification Involve a Trade-Off Between Technical Efficiency and Income Stability for Rural Farmers? Evidence from Zambia | 2020 |
| Nand M.M.; Bardsley D.K.; Suh J. | Addressing unavoidable climate change loss and damage: A case study from Fiji’s sugar industry | 2023 |
| Naz F., Saqib S.E., | Gender-based differences in flood vulnerability among men and women in the char farming households of Bangladesh | 2021 |
| Nchu I.N., Kimengsi J.N., Kapp G., | Diagnosing climate adaptation constraints in rural subsistence farming systems in Cameroon: Gender and institutional perspectives | 2019 |
| Ndlovu E., Prinsloo B., le Roux T., | Impact of climate change and variability on traditional farming systems: Farmers' perceptions from South-West, semi-arid Zimbabwe | 2020 |
| Nera, E; Paas, W; Reidsma, P; Paolini, G; Antonioli, F; Severini, S | Assessing the Resilience and Sustainability of a Hazelnut Farming System in Central Italy with a Participatory Approach | 2020 |
| Nettier B., Dobremez L., Coussy J.-L., Romagny T., | Attitudes of livestock farmers and sensitivity of livestock farming systems to drought conditions in the French Alps | 2010 |
| Nguyen H.Q., Tran D.D., Luan P.D.M.H., Ho L.H., Loan V.T.K., Anh Ngoc P.T., Quang N.D., Wyatt A., Sea W., | Socio-ecological resilience of mangrove-shrimp models under various threats exacerbated from salinity intrusion in coastal area of the Vietnamese Mekong Delta | 2020 |
| Nyong A.P., Ngankam T.M., Felicite T.L., | Enhancement of resilience to climate variability and change through agroforestry practices in smallholder farming systems in Cameroon | 2020 |
| Ocak Yetisgin S.; Önder H.; Şen U.; Piwczyński D.; Kolenda M.; Sitkowska B.; Yucel C. | Farmers’ Risk Perception on Climate Change: Transhumance vs. Semi-Intensive Sheep Production Systems in Türkiye | 2022 |
| Olumba C.N.; Ihemezie E.J.; Olumba C.C. | Climate change perception, adaptation strategies, and constraints amongst urban farmers in Anambra Metropolis, Nigeria | 2024 |
| Omerkhil N., Chand T., Valente D., Alatalo J.M., Pandey R., | Climate change vulnerability and adaptation strategies for smallholder farmers in Yangi Qala District, Takhar, Afghanistan | 2020 |
| Onyeneke R.U.; Amadi M.U.; Njoku C.L.; Osuji E.E. | Climate change perception and uptake of climate-smart agriculture in rice production in ebonyi state, nigeria | 2021 |
| Panta, B; Bhandari, T; Paudel, B | Soil erosion vulnerability and adaptation strategies in maize field of Sindhukhola sub-watershed region, Nepal | 2020 |
| Paul C., Weber M., Knoke T., | Agroforestry versus farm mosaic systems – Comparing land-use efficiency, economic returns and risks under climate change effects | 2017 |
| Pearson L.J.; Dare M. | Farmer pathways to sustainability in the face of water scarcity | 2021 |
| Perin L.; Enahoro D. | Foresight study on dairy farming systems in Central Kenya and north of Senegal | 2023 |
| Phelan D.C., Harrison M.T., Kemmerer E.P., Parsons D., | Management opportunities for boosting productivity of cool-temperate dairy farms under climate change | 2015 |
| Pinsard, C; Morais, TG; Domingos, T; Accatino, F; Teixeira, RFM | Strategies for future robust meat production and climate change mitigation under imported input constraints in Alentejo, Portugal | 2023 |
| Poelma T., Bayrak M.M., Van Nha D., Tran T.A., | Climate change and livelihood resilience capacities in the Mekong Delta: a case study on the transition to rice–shrimp farming in Vietnam’s Kien Giang Province | 2021 |
| Postigo J.C.; Guáqueta-Solórzano V.-E.; Castañeda E.; Ortiz-Guerrero C.E. | Adaptive Responses and Resilience of Small Livestock Producers to Climate Variability in the Cruz Verde-Sumapaz Páramo, Colombia | 2024 |
| Poulton P.L., Dalgliesh N.P., Vang S., Roth C.H., | Resilience of Cambodian lowland rice farming systems to future climate uncertainty | 2016 |
| Quach, AV; Murray, F; Morrison-Saunders, A | The vulnerability of shrimp farming income to climate change events A case study in Ca Mau, Vietnam | 2017 |
| Rai P.; Bajgai Y.; Rabgyal J.; Katwal T.B.; Delmond A.R. | Empirical Evidence of the Livelihood Vulnerability to Climate Change Impacts: A Case of Potato-Based Mountain Farming Systems in Bhutan | 2022 |
| Ranasinghe R.D.A.K.; Korale-Gedara P.M.; Weerasooriya S.A. | Climate change adaptation and adaptive capacities of dairy farmers: Evidence from village tank cascade systems in Sri Lanka | 2023 |
| Rankoana S.A. | Indigenous knowledge and innovative practices to cope with impacts of climate change on small-scale farming in Limpopo Province, South Africa | 2022 |
| Regassa N., Stoecker B.J., | Research article: Attitude and risk perceptions about climate change in farming communities in Southern Ethiopia | 2014 |
| Rezgui F.; Rosati A.; Lambarraa-Lehnhardt F.; Paul C.; Reckling M. | Assessing Mediterranean agroforestry systems: Agro-economic impacts of olive wild asparagus in central Italy | 2024 |
| Rigolot C., de Voil P., Douxchamps S., Prestwidge D., Van Wijk M., Thornton P.K., Rodriguez D., Henderson B., Medina D., Herrero M., | Interactions between intervention packages, climatic risk, climate change and food security in mixed crop–livestock systems in Burkina Faso | 2017 |
| Rodriguez D., Cox H., deVoil P., Power B., | A participatory whole farm modelling approach to understand impacts and increase preparedness to climate change in Australia | 2014 |
| Rodriguez, D; deVoil, P; Power, B; Cox, H; Crimp, S; Meinke, H | The intrinsic plasticity of farm businesses and their resilience to change. An Australian example | 2011 |
| Rotz, CA; Skinner, RH; Stoner, AMK; Hayhoe, K | EVALUATING GREENHOUSE GAS MITIGATION AND CLIMATE CHANGE ADAPTATION IN DAIRY PRODUCTION USING FARM SIMULATION | 2016 |
| Rufino M.C., Thornton P.K., Ng'ang'a S.K., Mutie I., Jones P.G., van Wijk M.T., Herrero M., | Transitions in agro-pastoralist systems of East Africa: Impacts on food security and poverty | 2013 |
| Sanogo K., Binam J., Bayala J., Villamor G.B., Kalinganire A., Dodiomon S., | Farmers’ perceptions of climate change impacts on ecosystem services delivery of parklands in southern Mali | 2017 |
| Sanou C.L.; Agodzo S.K.; Bessah E.; Antwi-Agyei P. | Assessing crop–livestock water productivity in mixed-farming systems across climatic zones of Burkina Faso | 2023 |
| Schaap B.F., Reidsma P., Verhagen J., Wolf J., van Ittersum M.K., | Participatory design of farm level adaptation to climate risks in an arable region in The Netherlands | 2013 |
| Schmitt Olabisi L., Liverpool-Tasie S., Rivers L., III, Ligmann-Zielinska A., Du J., Denny R., Marquart-Pyatt S., Sidibé A., | Using participatory modeling processes to identify sources of climate risk in West Africa | 2018 |
| Scott J.F., Cacho O.J., Scott J.M., | Economic risk analysis of different livestock management systems | 2013 |
| Shah A.A.; Khan N.A.; Gong Z.; Ahmad I.; Naqvi S.A.A.; Ullah W.; Karmaoui A. | Farmers’ perspective towards climate change vulnerability, risk perceptions, and adaptation measures in Khyber Pakhtunkhwa, Pakistan | 2023 |
| Shantharaju A.; Islam M.A.; Kath J.M.; Mushtaq S.; Muniyappa A.; Singh-Peterson L. | Understanding Constraints and Enablers of Climate Risk Management Strategies: Evidence from Smallholder Dairy Farmers in Regional South India | 2024 |
| Shrestha R.P., Nepal N., | An assessment by subsistence farmers of the risks to food security attributable to climate change in Makwanpur, Nepal | 2016 |
| Shrestha S., Barratt A., Fox N.J., Vosough Ahmadi B., Hutchings M.R., | Financial Impacts of Liver Fluke on Livestock Farms Under Climate Change–A Farm Level Assessment | 2020 |
| Simelton E., Dam B.V., Catacutan D., | Trees and agroforestry for coping with extreme weather events: experiences from northern and central Viet Nam | 2015 |
| Simelton E., Quinn C.H., Batisani N., Dougill A.J., Dyer J.C., Fraser E.D.G., Mkwambisi D., Sallu S., Stringer L.C., | Is rainfall really changing? Farmers' perceptions, meteorological data, and policy implications | 2013 |
| Singh A.K., Das B., Mali S.S., Bhavana P., Shinde R., Bhatt B.P., | Intensification of rice-fallow cropping systems in the Eastern Plateau region of India: diversifying cropping systems and climate risk mitigation | 2020 |
| Soltani L.; Mellah T. | Exploring farmers’ adaptation strategies to water shortage under climate change in the Tunisian semi-arid region | 2023 |
| Son H.N., Kingsbury A., Hoa H.T., | Indigenous knowledge and the enhancement of community resilience to climate change in the Northern Mountainous Region of Vietnam | 2021 |
| Sorvali, J; Kaseva, J; Peltonen-Sainio, P | Farmer views on climate change-a longitudinal study of threats, opportunities and action | 2021 |
| Souissi, I; Boisson, JM; Mekki, I; Therond, O; Flichman, G; Wery, J; Belhouchette, H | Impact assessment of climate change on farming systems in the South Mediterranean area: a Tunisian case study | 2018 |
| Srinidhi A.; Smolenaars W.; Werners S.E.; Hegde S.; Rajapure G.; Meuwissen M.P.M.; Ludwig F. | Critical climate-stress moments for semi-arid farming systems in India | 2024 |
| Srinidhi A.; Werners S.E.; Dadas D.; D’Souza M.; Ludwig F.; Meuwissen M.P.M. | Retrospective climate resilience assessment of semi-arid farming systems in India | 2024 |
| Stewart-Koster B., Dieu Anh N., Burford M.A., Condon J., Qui N.V., Hiep L.H., Bay D.V., Sammut J., | Expert based model building to quantify risk factors in a combined aquaculture-agriculture system | 2017 |
| Sujakhu N.M., Ranjitkar S., Niraula R.R., Pokharel B.K., Schmidt-Vogt D., Xu J., | Farmers' perceptions of and adaptations to changing climate in the Melamchi Valley of Nepal | 2016 |
| Tan D.N. | Analysing the livelihood vulnerability of shrimp farmers to climate change: A case study in Tra Vinh province, Vietnam | 2022 |
| Taremwa N.K., Gashumba D., Butera A., Ranganathan T., | Climate Change Adaptation in Rwanda through Indigenous Knowledge Practice | 2016 |
| Tibesigwa, B; Visser, M; Turpie, J | Climate change and South Africa's commercial farms: an assessment of impacts on specialised horticulture, crop, livestock and mixed farming systems | 2017 |
| Toni F., Holanda Jr. E., | The effects of land tenure on vulnerability to droughts in Northeastern Brazil | 2008 |
| Touch V., Martin R.J., Scott F., Cowie A., Liu D.L., | Climate change impacts on rainfed cropping production systems in the tropics and the case of smallholder farms in North-west Cambodia | 2017 |
| Tran T.A.; Cook B.R. | Water retention for agricultural resilience in the Vietnamese Mekong Delta: towards integrated ‘grey–green’ solutions | 2024 |
| Tui S.H.-K., Descheemaeker K., Valdivia R.O., Masikati P., Sisito G., Moyo E.N., Crespo O., Ruane A.C., Rosenzweig C., | Climate change impacts and adaptation for dryland farming systems in Zimbabwe: a stakeholder-driven integrated multi-model assessment | 2021 |
| Tun Oo A., Van Huylenbroeck G., Speelman S., | Determining factors for the application of climate change adaptation strategies among farmers in Magwe District, dry zone region of Myanmar | 2017 |
| Valliant J.C.D., Bruce A.B., Houser M., Dickinson S.L., Farmer J.R., | Product Diversification, Adaptive Management, and Climate Change: Farming and Family in the U.S. Corn Belt | 2021 |
| Varadan, RJ; Mamidanna, S; Kumar, S; Ahmed, SKZ; Jaisankar, | Technology, infrastructure and enterprise trade-off: Strengthening smallholder farming systems in Tamil Nadu State of India for sustainable income and food security | 2022 |
| Vigani, M; Kathage, J | TO RISK OR NOT TO RISK? RISK MANAGEMENT AND FARM PRODUCTIVITY | 2019 |
| Wetende E., Olago D., Ogara W., | Perceptions of climate change variability and adaptation strategies on smallholder dairy farming systems: Insights from Siaya Sub-County of Western Kenya | 2018 |
| Williams N.E., Carrico A.R., Edirisinghe I., Jayamini Champika P.A., | Assessing the Impacts of Agrobiodiversity Maintenance on Food Security Among Farming Households in Sri Lanka’s Dry Zone | 2018 |
| Williams P.A., Crespo O., Abu M., | Assessing vulnerability of horticultural smallholders’ to climate variability in Ghana: applying the livelihood vulnerability approach | 2020 |
| Williams T.G., Guikema S.D., Brown D.G., Agrawal A., | Resilience and equity: Quantifying the distributional effects of resilience-enhancing strategies in a smallholder agricultural system | 2020 |
| Yeleliere E.; Antwi-Agyei P.; Guodaar L. | Farmers response to climate variability and change in rainfed farming systems: Insight from lived experiences of farmers | 2023 |
| Yessoufou A.N.-D.; Kumar S.; Houessionon P.; Worou O.N.; Wane A.; Whitbread A. | Vulnerability and resilience in the face of climate changes in Senegal's drylands: measurement at the household level and determinant assessment | 2024 |
| Young M.; Young J.; Kingwell R.S.; Vercoe P.E. | Identifying high-value tactical livestock decisions on a mixed enterprise farm in a variable environment | 2024 |
| Zinyemba, C; Archer, E; Rother, HA | Climate Change, Pesticides and Health: Considering the Risks and Opportunities of Adaptation for Zimbabwean Smallholder Cotton Growers | 2021 |
